# Supplementary material for: Leaky Gut Biomarkers as Predictors of Depression and Suicidal Risk: A Systematic Review and Meta-Analysis
Source: Diagnostics (Basel). 2025 Jul 1;15(13):1683. doi: 10.3390/diagnostics15131683 (PMC12249198; doi:10.3390/diagnostics15131683)
Supplement: Supplementary file 1 [file diagnostics-15-01683-s001.zip › Supplement S1 LGM 30.06.pdf]

Leaky Gut Biomarkers as Predictors of Depression and Suicidal Risk: A Systematic Review and Meta-Analysis – Supplement S1

Appendix S1 – All Biomarkers Investigated in The Systematic Review And Their Role In Leaky Gut Syndrome

Table S1. Complete description of all biomarkers investigated in the systematic review

Category	**Biomarker**	**Description**
Structural Proteins	Zonulin	Zonulin is an endogenous human protein that plays a critical role in regulating intestinal permeability by modulating tight junctions between epithelial cells lining the intestinal mucosa. By dynamically influencing the integrity of these intercellular junctions, zonulin controls the selective permeability of the intestinal barrier, either facilitating or restricting the passage of molecules from the intestinal lumen into systemic circulation. This regulatory mechanism is essential for balancing nutrient absorption, immune surveillance, and defence against harmful antigens or pathogens (1).
	Occludin	Occludin is a key protein of intestinal tight junctions, crucial for regulating intestinal barrier permeability. Alterations in its levels can compromise barrier integrity, allowing the translocation of harmful molecules and pathogens and potentially triggering systemic inflammation. Measurement of occludin in biological samples, such as plasma or intestinal tissues, serves as a marker of barrier status, with reduced levels indicating barrier dysfunction (2).
	Intestinal Fatty Acid Binding Protein (I-FABP)	I-FABP is a cytoplasmic protein predominantly expressed by enterocytes in the small intestine. As a member of the fatty acid-binding protein family, it plays a crucial role in binding, transporting, and metabolising long-chain fatty acids, contributing to lipid homeostasis and energy balance in the intestinal mucosa. Highly concentrated in enterocytes, I-FABP serves as a sensitive biomarker of intestinal epithelial damage, with its release into the bloodstream or urine indicating compromised mucosal integrity (3).
Immune Proteins	Calprotectin	Calprotectin is a protein predominantly produced by neutrophils during inflammatory responses. It is released into tissues and faeces during active inflammation, with elevated faecal levels indicating neutrophil recruitment to the intestine. This is often associated with a compromised epithelial barrier, making calprotectin a valuable biomarker for intestinal inflammation and barrier dysfunction (4).
	Human β-defensin 2 (hBD-2)	hBD-2 is an antimicrobial protein produced by epithelial cells in response to infections and inflammation, playing a vital role in innate immunity. It exhibits broad-spectrum activity against bacteria, fungi, and viruses, aiding in pathogen elimination while modulating the immune response by recruiting immune cells such as monocytes and neutrophils. Additionally, hBD-2 supports epithelial barrier repair and strengthens tight junctions, contributing to intestinal integrity. Elevated levels in faeces or plasma are observed in chronic inflammatory bowel diseases, whereas insufficient production is linked to barrier dysfunction, bacterial translocation, and systemic inflammation (5).
	Alpha-1 antitrypsin (A-1-AT)	A-1-AT is a protein that serves as a crucial protease inhibitor, protecting tissues from proteolytic enzymes released during inflammatory processes. Under normal conditions, A1AT helps preserve the integrity of the intestinal epithelial barrier through its anti-inflammatory and cytoprotective properties. It also modulates the immune response by inhibiting the release of pro-inflammatory cytokines, which could otherwise compromise barrier function (6).
	Lipopolysaccharide-binding protein (LBP)	LBP is primarily produced by the liver and belongs to the acute-phase protein family. It plays a crucial role in the immune response by recognising lipopolysaccharides (LPS) from Gram-negative bacteria. LBP binds to LPS and facilitates their transfer to innate immune receptors, such as CD14 and Toll-like receptor 4 (TLR4), initiating an inflammatory response. Elevated plasma levels of LBP serve as a biomarker of increased intestinal permeability, indicating excessive LPS translocation into the bloodstream and systemic inflammation driven by bacterial translocation (7).

	Soluble CD14 (sCD14)	sCD14 is a circulating form of the CD14 protein found in plasma and biological fluids, playing a key role in modulating the inflammatory response. It binds to lipopolysaccharides (LPS) in circulation, neutralising them and regulating their interaction with Toll-like receptor 4 (TLR4), which triggers the inflammatory cascade. Elevated plasma sCD14 levels serve as an indirect marker of impaired intestinal permeability and increased circulating LPS, indicating systemic immune activation (8).
	Lactoferrin	Lactoferrin is a multifunctional protein primarily produced by neutrophils, epithelial cells of mucosal surfaces, and exocrine glands (such as in milk, saliva, and mucus). It plays a crucial role in protecting the intestinal barrier by strengthening tight junctions between epithelial cells, modulating inflammation, and counteracting dysbiosis. In cases of intestinal barrier dysfunction (commonly referred to as "leaky gut"), fecal lactoferrin levels increase as a response to barrier impairment and neutrophil recruitment, reflecting ongoing inflammation and tissue damage (9).
	Antibodies to endotoxins	Immunoglobulins are generated by the immune system in response to the translocation of endotoxins (including Lipopolysaccharide, LPS), a collective term for components of the external membrane of the cell envelope of Gram-negative bacteria, from the intestinal lumen into the systemic circulation. The detection of anti-endotoxin antibodies (e.g., Ig against LPS) in the bloodstream serves as a potential biomarker suggestive of increased intestinal permeability and systemic endotoxin exposure. Endotoxins are known to trigger neuroinflammation through the activation of microglial cells, which are the resident immune cells of the central nervous system. The presence of LPS in the bloodstream can lead to the release of pro-inflammatory cytokines, contributing to a cascade of inflammatory responses in the brain (10).
**Bacterial Endotoxins**	Lipopolysaccharides (LPS) / endotoxins	Endotoxins (11) are a diverse group of molecules, with Lipopolysaccharide (LPS) being the most well-known and studied. LPS is found in the outer membrane of Gram-negative bacteria, and it consists of a lipid component (lipid A), which is responsible for its toxicity, and a carbohydrate component (core polysaccharide and O-antigen). While LPS is the primary endotoxin in this context, other endotoxins can also have similar effects. For instance, lipoteichoic acids (LTA), found in the cell walls of Gram-positive bacteria, and peptidoglycan fragments are also considered endotoxins that can stimulate immune responses.<br>Produced primarily by Gram-negative bacteria within the gut microbiota, LPS and other endotoxins can translocate across a compromised intestinal barrier, a condition often referred to as intestinal hyperpermeability. Once in the bloodstream, endotoxins trigger the activation of the immune system, particularly through the recognition by Toll-like receptors (TLRs), such as TLR4 for LPS. This activation initiates a cascade of pro-inflammatory responses, leading to systemic inflammation. These endotoxins have been implicated in various chronic diseases, including metabolic disorders, cardiovascular diseases, and neuroinflammation, as they can reach distant organs, including the brain, where they may exacerbate inflammation and contribute to conditions like neurodegenerative diseases (12).

Appendix S2 – PRISMA 2020 Checklist

Section and Topic	Item #	Checklist item	Location where item is reported
**TITLE**			
Title	1	Identify the report as a systematic review.	Pag. 1
**ABSTRACT**			
Abstract	2	See the PRISMA 2020 for Abstracts checklist.	Pag. 1
**INTRODUCTION**			
Rationale	3	Describe the rationale for the review in the context of existing knowledge.	Pag. 2-5
Objectives	4	Provide an explicit statement of the objective(s) or question(s) the review addresses.	Pag. 2-5
**METHODS**			
Eligibility criteria	5	Specify the inclusion and exclusion criteria for the review and how studies were grouped for the syntheses.	Pag. 5<br>Supplement 1
Information sources	6	Specify all databases, registers, websites, organisations, reference lists and other sources searched or consulted to identify studies. Specify the date when each source was last searched or consulted.	Pag. 5
Search strategy	7	Present the full search strategies for all databases, registers and websites, including any filters and limits used.	Pag. 5-6<br>Supplement 1
Selection process	8	Specify the methods used to decide whether a study met the inclusion criteria of the review, including how many reviewers screened each record and each report retrieved, whether they worked independently, and if applicable, details of automation tools used in the process.	Pag. 6<br>Supplement 1
Data collection process	9	Specify the methods used to collect data from reports, including how many reviewers collected data from each report, whether they worked independently, any processes for obtaining or confirming data from study investigators, and if applicable, details of automation tools used in the process.	Pag. 5-7
Data items	10a	List and define all outcomes for which data were sought. Specify whether all results that were compatible with each outcome domain in each study were sought (e.g. for all measures, time points, analyses), and if not, the methods used to decide which results to collect.	Pag. 5-7
	10b	List and define all other variables for which data were sought (e.g. participant and intervention characteristics, funding sources). Describe any assumptions made about any missing or unclear information.	Pag. 5-9
Study risk of bias assessment	11	Specify the methods used to assess risk of bias in the included studies, including details of the tool(s) used, how many reviewers assessed each study and whether they worked independently, and if applicable, details of automation tools used in the process.	Pag. 6<br>Supplement 1
Effect measures	12	Specify for each outcome the effect measure(s) (e.g. risk ratio, mean difference) used in the synthesis or presentation of results.	Pag. 7
Synthesis methods	13a	Describe the processes used to decide which studies were eligible for each synthesis (e.g. tabulating the study intervention characteristics and comparing against the planned groups for each synthesis (item #5)).	Pag. 5-7
	13b	Describe any methods required to prepare the data for presentation or synthesis, such as handling of missing summary statistics, or data conversions.	Pag. 5-7
	13c	Describe any methods used to tabulate or visually display results of individual studies and syntheses.	Pag. 7-9
	13d	Describe any methods used to synthesize results and provide a rationale for the choice(s). If meta-analysis was performed, describe the	Pag. 7-9

Section and Topic	Item #	Checklist item	Location where item is reported
		model(s), method(s) to identify the presence and extent of statistical heterogeneity, and software package(s) used.	
	13e	Describe any methods used to explore possible causes of heterogeneity among study results (e.g. subgroup analysis, meta-regression).	Pag. 22 Supplement 1
	13f	Describe any sensitivity analyses conducted to assess robustness of the synthesized results.	Pag. 22 Supplement 1
Reporting bias assessment	14	Describe any methods used to assess risk of bias due to missing results in a synthesis (arising from reporting biases).	Pag. 22 Supplement 1
Certainty assessment	15	Describe any methods used to assess certainty (or confidence) in the body of evidence for an outcome.	Pag. 22 Supplement 1
**RESULTS**			
Study selection	16a	Describe the results of the search and selection process, from the number of records identified in the search to the number of studies included in the review, ideally using a flow diagram.	Pag. 7-22
	16b	Cite studies that might appear to meet the inclusion criteria, but which were excluded, and explain why they were excluded.	Supplement 3
Study characteristics	17	Cite each included study and present its characteristics.	Pag. 7-22
Risk of bias in studies	18	Present assessments of risk of bias for each included study.	Pag. 7-22 Supplement 1
Results of individual studies	19	For all outcomes, present, for each study: (a) summary statistics for each group (where appropriate) and (b) an effect estimate and its precision (e.g. confidence/credible interval), ideally using structured tables or plots.	Pag. 7-22
Results of syntheses	20a	For each synthesis, briefly summarise the characteristics and risk of bias among contributing studies.	Pag. 7-22
	20b	Present results of all statistical syntheses conducted. If meta-analysis was done, present for each the summary estimate and its precision (e.g. confidence/credible interval) and measures of statistical heterogeneity. If comparing groups, describe the direction of the effect.	Pag. 7-22 Supplement 1
	20c	Present results of all investigations of possible causes of heterogeneity among study results.	Pag. 7-22 Supplement 1
	20d	Present results of all sensitivity analyses conducted to assess the robustness of the synthesized results.	Pag. 7-22 Supplement 1
Reporting biases	21	Present assessments of risk of bias due to missing results (arising from reporting biases) for each synthesis assessed.	Pag. 7-22 Supplement 1
Certainty of evidence	22	Present assessments of certainty (or confidence) in the body of evidence for each outcome assessed.	Pag. 7-22 Supplement 1
**DISCUSSION**			
Discussion	23a	Provide a general interpretation of the results in the context of other evidence.	Pag. 22-25
	23b	Discuss any limitations of the evidence included in the review.	Pag. 25

Section and Topic	Item #	Checklist item	Location where item is reported
	23c	Discuss any limitations of the review processes used.	Pag. 25
	23d	Discuss implications of the results for practice, policy, and future research.	Pag. 25-26
**OTHER INFORMATION**			
Registration and protocol	24a	Provide registration information for the review, including register name and registration number, or state that the review was not registered.	Pag. 7
	24b	Indicate where the review protocol can be accessed, or state that a protocol was not prepared.	Pag. 7
	24c	Describe and explain any amendments to information provided at registration or in the protocol.	Pag. 7
Support	25	Describe sources of financial or non-financial support for the review, and the role of the funders or sponsors in the review.	Pag. 26
Competing interests	26	Declare any competing interests of review authors.	Pag. 26
Availability of data, code and other materials	27	Report which of the following are publicly available and where they can be found: template data collection forms; data extracted from included studies; data used for all analyses; analytic code; any other materials used in the review.	Pag. 26

From: Page MJ, McKenzie JE, Bossuyt PM, Boutron I, Hoffmann TC, Mulrow CD, et al. The PRISMA 2020 statement: an updated guideline for reporting systematic reviews. BMJ 2021;372:n71. doi: 10.1136/bmj.n71. This work is licensed under CC BY 4.0. To view a copy of this license, visit https://creativecommons.org/licenses/by/4.0/

Appendix S3 – Search String and Inclusion Criteria

PUBMED

Search:

(depressive disorder[MeSH Terms] OR depression[MeSH Terms] OR depressive disorder, major[MeSH Terms] OR depress*[Title/Abstract] OR depression[Title/Abstract] OR depressive disorder[Title/Abstract] OR depressive state[Title/Abstract] OR depressive symptom[Title/Abstract] OR depressed[Title/Abstract] OR depressive[Title/Abstract] OR MDD[Title/Abstract] OR major depressive disorder[Title/Abstract] OR dysthymia[Title/Abstract] OR melancholia[Title/Abstract] OR suicide[MeSH Terms] OR suicid*[Title/Abstract] OR suicidal*[Title/Abstract] OR suicidality[Title/Abstract] OR suicidal ideation[Title/Abstract] OR suicidal attempt[Title/Abstract] OR self-killing[Title/Abstract] OR self-harm[Title/Abstract] OR self-injury[Title/Abstract] AND (humans[Filter]))

AND

(Zonulin[Title/Abstract] OR Lipopolysaccharide-binding protein[Title/Abstract] OR Lipopolysaccharide binding protein[Title/Abstract] OR lipopolysaccharide[Title/Abstract] OR LBP[Title/Abstract] OR Soluble CD14[Title/Abstract] OR sCD14[Title/Abstract] OR Fatty acid-binding protein 2[Title/Abstract] OR FABP2[Title/Abstract] OR Intestinal fatty-acid binding protein[Title/Abstract] OR intestinal fatty acid binding protein[Title/Abstract] OR I-FABP[Title/Abstract] OR Calprotectin[Title/Abstract] OR Intestinal alkaline phosphatase[Title/Abstract] OR IAP[Title/Abstract] OR Bactericidal/permeability-increasing protein[Title/Abstract] OR BPI[Title/Abstract] OR alpha-1-antitrypsin[Title/Abstract] OR A-1-AT[Title/Abstract] OR Peptidoglycan[Title/Abstract] OR OR Endotoxins[Title/Abstract] OR Claudin[Title/Abstract] OR Occludin tight junction protein[Title/Abstract] OR CLDN-1[Title/Abstract] OR OCLN[Title/Abstract] OR anti-Saccharomyces cerevisiae antibodies[Title/Abstract] OR IgA Saccharomyces cerevisiae[Title/Abstract] OR IgM Saccharomyces cerevisiae[Title/Abstract] OR IgG gram-negative bacteria[Title/Abstract] OR IgA gram-negative bacteria[Title/Abstract] OR IgM gram-negative bacteria[Title/Abstract] OR OR ASCA[Title/Abstract] OR IgG LPS[Title/Abstract] OR IgA LPS[Title/Abstract] OR IgM LPS[Title/Abstract] AND (humans[Filter]))

Filters: Humans, from 2000 - 2024 Sort by: Most Recent

EMBASE

(depressive disorder/exp OR depression/exp OR major depression/exp OR depress*:ti,ab OR depression:ti,ab OR depressive disorder:ti,ab OR depressive state:ti,ab OR depressive symptom:ti,ab OR depressed:ti,ab OR depressive:ti,ab OR MDD:ti,ab OR major depressive disorder:ti,ab OR dysthymia:ti,ab OR melancholia:ti,ab OR suicide/exp OR suicid*:ti,ab OR suicidal*:ti,ab OR suicidality:ti,ab OR suicidal ideation:ti,ab OR suicidal attempt:ti,ab OR self-killing:ti,ab OR self-harm:ti,ab OR self-injury:ti,ab)

AND

(zonulin:ti,ab OR lipopolysaccharide-binding protein:ti,ab OR lipopolysaccharide binding protein:ti,ab OR lipopolysaccharide:ti,ab OR LBP:ti,ab OR soluble CD14:ti,ab OR sCD14:ti,ab OR

fatty acid-binding protein 2:ti,ab OR FABP2:ti,ab OR intestinal fatty-acid binding protein:ti,ab OR intestinal fatty acid binding protein:ti,ab OR I-FABP:ti,ab OR calprotectin:ti,ab OR intestinal alkaline phosphatase:ti,ab OR IAP:ti,ab OR bactericidal/permeability-increasing protein:ti,ab OR BPI:ti,ab OR alpha-1-antitrypsin:ti,ab OR A-1-AT:ti,ab OR peptidoglycan:ti,ab OR endotoxins:ti,ab OR claudin:ti,ab OR occludin tight junction protein:ti,ab OR CLDN-1:ti,ab OR OCLN:ti,ab OR anti-Saccharomy

SCOPUS

(TITLE-ABS-KEY(depressive disorder) OR TITLE-ABS-KEY(depression) OR TITLE-ABS-KEY(depressive disorder, major) OR TITLE-ABS-KEY(depress*) OR TITLE-ABS-KEY(depression) OR TITLE-ABS-KEY(depressive disorder) OR TITLE-ABS-KEY(depressive state) OR TITLE-ABS-KEY(depressive symptom) OR TITLE-ABS-KEY(depressed) OR TITLE-ABS-KEY(depressive) OR TITLE-ABS-KEY(MDD) OR TITLE-ABS-KEY(major depressive disorder) OR TITLE-ABS-KEY(dysthymia) OR TITLE-ABS-KEY(melancholia) OR TITLE-ABS-KEY(suicide) OR TITLE-ABS-KEY(suicid*) OR TITLE-ABS-KEY(suicidal*) OR TITLE-ABS-KEY(suicidality) OR TITLE-ABS-KEY(suicidal ideation) OR TITLE-ABS-KEY(suicidal attempt) OR TITLE-ABS-KEY(self-killing) OR TITLE-ABS-KEY(self-harm) OR TITLE-ABS-KEY(self-injury))

AND

(TITLE-ABS-KEY(zonulin) OR TITLE-ABS-KEY(lipopolysaccharide-binding protein) OR TITLE-ABS-KEY(lipopolysaccharide binding protein) OR TITLE-ABS-KEY(lipopolysaccharide) OR TITLE-ABS-KEY(LBP) OR TITLE-ABS-KEY(soluble CD14) OR TITLE-ABS-KEY(sCD14) OR TITLE-ABS-KEY(fatty acid-binding protein 2) OR TITLE-ABS-KEY(FABP2) OR TITLE-ABS-KEY(intestinal fatty-acid binding protein) OR TITLE-ABS-KEY(intestinal fatty acid binding protein) OR TITLE-ABS-KEY(I-FABP) OR TITLE-ABS-KEY(calprotectin) OR TITLE-ABS-KEY(intestinal alkaline phosphatase) OR TITLE-ABS-KEY(IAP) OR TITLE-ABS-KEY(bactericidal/permeability-increasing protein) OR TITLE-ABS-KEY(BPI) OR TITLE-ABS-KEY(alpha-1-antitrypsin) OR TITLE-ABS-KEY(A-1-AT) OR TITLE-ABS-KEY(peptidoglycan) OR TITLE-ABS-KEY(endotoxins) OR TITLE-ABS-KEY(claudin) OR TITLE-ABS-KEY(occludin tight junction protein) OR TITLE-ABS-KEY(CLDN-1) OR TITLE-ABS-KEY(OCLN) OR TITLE-ABS-KEY(anti-Saccharomyces cerevisiae antibodies) OR TITLE-ABS-KEY(IgA Saccharomyces cerevisiae) OR TITLE-ABS-KEY(IgM Saccharomyces cerevisiae) OR TITLE-ABS-KEY(IgG gram-negative bacteria) OR TITLE-ABS-KEY(IgA gram-negative bacteria) OR TITLE-ABS-KEY(IgM gram-negative bacteria) OR TITLE-ABS-KEY(Ig-ASCA) OR TITLE-ABS-KEY(ASCA) OR TITLE-ABS-KEY(IgG LPS) OR TITLE-ABS-KEY(IgA LPS) OR TITLE-ABS-KEY(IgM LPS))

AND (LIMIT-TO(DOCTYPE, "ar"))

COCHRANE LIBRARY

ID	Search
#1	“depressive disorder”:ti,ab,kw (Word variations have been searched)
#2	“depression”:ti,ab,kw (Word variations have been searched)
#3	“major depression”:ti,ab,kw (Word variations have been searched)
#4	“depress*”:ti,ab,kw (Word variations have been searched)
#5	“depression”:ti,ab,kw (Word variations have been searched)
#6	“depressive disorder”:ti,ab,kw (Word variations have been searched)

#7 “depressive state”:ti,ab,kw (Word variations have been searched)
#8 “depressive symptom”:ti,ab,kw (Word variations have been searched)
#9 “depressed”:ti,ab,kw (Word variations have been searched)
#10 “depressive”:ti,ab,kw (Word variations have been searched)
#11 “MDD”:ti,ab,kw (Word variations have been searched)
#12 “major depressive disorder”:ti,ab,kw (Word variations have been searched)
#13 “dysthymia”:ti,ab,kw (Word variations have been searched)
#14 “melancholia”:ti,ab,kw (Word variations have been searched)
#15 “suicide”:ti,ab,kw (Word variations have been searched)
#16 “suicide*”:ti,ab,kw (Word variations have been searched)
#17 “suicidal*”:ti,ab,kw (Word variations have been searched)
#18 “suicidality”:ti,ab,kw (Word variations have been searched)
#19 “suicidal ideation”:ti,ab,kw (Word variations have been searched)
#20 “suicidal attempt”:ti,ab,kw (Word variations have been searched)
#21 “self-killing”:ti,ab,kw (Word variations have been searched)
#22 “self-harm”:ti,ab,kw (Word variations have been searched)
#23 “self-injury”:ti,ab,kw (Word variations have been searched)
#24 #1 OR #2 OR #3 OR #4 OR #5 OR #6 OR #7 OR #8 OR #9 OR #10 OR #11OR #12 OR #13 OR #14 OR #15 OR #16 OR #17 OR #18 OR #19 OR #20 OR #21 OR #22 OR #23
#25 “zonulin”:ti,ab,kw (Word variations have been searched)
#26 “lipopolysaccharide-binding protein”:ti,ab,kw (Word variations have been searched)
#27 “lipopolysaccharide”:ti,ab,kw (Word variations have been searched)
#28 “LBP”:ti,ab,kw (Word variations have been searched)
#29 “soluble CD14”:ti,ab,kw (Word variations have been searched)
#30 “sCD14”:ti,ab,kw (Word variations have been searched)
#31 “fatty acid-binding protein 2”:ti,ab,kw (Word variations have been searched)
#32 “FABP2”:ti,ab,kw (Word variations have been searched)
#33 “intestinal fatty-acid binding protein”:ti,ab,kw (Word variations have been searched)
#34 “I-FABP”:ti,ab,kw (Word variations have been searched)
#35 “calprotectin”:ti,ab,kw (Word variations have been searched)
#36 “intestinal alkaline phosphatase”:ti,ab,kw (Word variations have been searched)
#37 “IAP”:ti,ab,kw (Word variations have been searched)
#38 “bactericidal/permeability-increasing protein”:ti,ab,kw (Word variations have been searched)
#39 “BPI”:ti,ab,kw (Word variations have been searched)
#40 “alpha-1-antitrypsin”:ti,ab,kw (Word variations have been searched)
#41 “A-1-AT”:ti,ab,kw (Word variations have been searched)
#42 “peptidoglycan”:ti,ab,kw (Word variations have been searched)
#43 “endotoxins”:ti,ab,kw (Word variations have been searched)
#44 “claudin”:ti,ab,kw (Word variations have been searched)
#45 “occludin”:ti,ab,kw (Word variations have been searched)
#46 “tight junction protein”:ti,ab,kw (Word variations have been searched)
#47 “CLDN-1”:ti,ab,kw (Word variations have been searched)
#48 “OCLN”:ti,ab,kw (Word variations have been searched)
#49 “anti-Saccharomy”:ti,ab,kw (Word variations have been searched)
#50 #24 OR #25 OR #26 OR #27 OR #28 OR #29 OR #30 OR #31 OR #32 OR #33 OR #34 OR #35 OR #36 OR #37 OR #38 OR #39 OR #40 OR #41 OR #42 OR #43 OR #44 OR #45 OR #46 OR #47 OR #48 OR #49
#51 #24 AND #50 in Trials

Inclusion criteria

- Case-control studies reporting data on easily accessible biomarkers (e.g., blood, serum, plasma, faecal) for intestinal permeability and bacterial translocation will be included. Studies must include participants with a diagnosis of mood disorder, according to internationally validated criteria, and with the actual presence of depressive symptoms. Studies reporting data on in- or out-patients at any stage of illness, irrespective of their medication status, will be included, as well as studies involving patients with a recent suicide attempt, current suicidal ideation, or high levels of suicidality as measured by standardized assessment tools.

- Studies investigating the relationship between easily accessible biomarkers (e.g., serum, plasma, faecal) for intestinal permeability and depressive symptoms measured with standardised scales will be included. Studies must report correlation values between depressive scores and the proxy biomarkers investigated.

- Studies published in English.

Exclusion criteria

- Reviews, studies published in conference abstracts, letters to the editor;

- Articles published before January 2000. Applying a pre-2000 cutoff ensures inclusion of studies with standardized and reliable biomarker assays, consistent diagnostic criteria for depression, and methodological rigor. Since the gut-brain axis concept and advanced analytical techniques gained prominence mostly after 2000, this filter helps focus on relevant, high-quality research and improves comparability across studies.

Appendix S4 – Quality of evidence extended

1) Leaky gut biomarkers in depression and suicidal behavior" - Ohlsson et al., 2019 (13)

Appraisal Point	Positive/Methodologically Sound	Negative/Relatively Poor Methodology	Unknowns	Risk of Bias
Focus of the study	Clearly addresses the link between intestinal permeability and mental health.			Low
Methodological appropriateness	The cross-sectional design is appropriate for exploring associations.		Limited by its inability to infer causality.	Low
Sample recruitment	Systematic recruitment of participants.		Potential selection bias not fully discussed.	Low
Measurement accuracy	Biomarkers measured using validated ELISA kits.		Potential variability in biomarker processing not explicitly detailed.	Low
Data collection adequacy	Demographic and clinical data were rigorously collected.		Key confounders like diet, smoking, and alcohol were not addressed.	Low
Sample size sufficiency		Small sample size (54 rSA, 13 nsMDD, 17 HC); imbalance across groups. Insufficient for more complex or subgroup analyses.		Medium
Presentation of results	Results presented clearly (medians, IQR, ANOVA, correlations).	No sensitivity analysis to confirm robustness of findings.		Low
Data analysis rigor	Appropriate use of ANCOVA and adjustments for BMI and medications.		Further confounders not addressed.	Low
Clarity of findings	Findings clearly linked to the "leaky gut" hypothesis.		Biological mechanisms behind associations are speculative.	Low
Applicability to local population	Relevant for populations in similar healthcare settings.	Limited generalizability due to single-center design in Sweden.	Broader applicability to non-European populations is unclear.	Medium/ Can't Tell
Research value	Highlights innovative connections between gut permeability and mental health.	Small sample size. Long-term clinical implications require further validation.		Can't Tell

2) Abnormal Distribution and Function of Circulating Monocytes and Enhanced Bacterial Translocation in Major Depressive Disorder -Alvarez-Mon et al., 2019 (14)

Appraisal Point	Positive/Methodologically Sound	Negative/Relatively Poor Methodology	Unknowns	Risk of Bias
Focus of the study	Clearly investigates monocyte alterations and bacterial translocation in MDD.			Low
Methodological appropriateness	Cross-sectional design aligns with the exploratory objectives.		Lack of longitudinal data	Low

			for causal inference.	
Sample recruitment	Age- and sex-matched control group included.		Details of selection criteria for controls not fully explained.	Low
Measurement accuracy	Cytokines and biomarkers measured using validated ELISA and flow cytometry.			Low
Data collection adequacy	Comprehensive data on cytokines, monocyte subsets, and bacterial translocation.		Dietary and lifestyle factors not assessed as confounders.	Low
Sample size sufficiency	Adequate for identifying associations.	Small sample size (22 MDD patients, 14 controls).	limited for subgroup analysis.	Medium
Presentation of results	Results presented as mean ± SD; median (IQR) with statistical tests.		Lack of sensitivity analysis for robustness.	Low
Data analysis rigor	Proper use of Mann-Whitney U test and Spearman's correlation.		Interaction effects not explored.	Low
Clarity of findings	Findings clearly suggest a link between monocyte changes and bacterial translocation in MDD.		Mechanistic explanation remains speculative.	Low
Applicability to local population	Relevant for similar clinical populations.	Single-center design limits generalizability.	Broader applicability to diverse ethnic groups unclear.	Medium/ Can't Tell
Research value	Advances understanding of gut-brain axis and immune alterations in MDD.	Small sample size. Long-term clinical implications require further validation.		Can't Tell

3) Blunted Expansion of Regulatory T Lymphocytes Is Associated With Increased Bacterial Translocation in Patients With Major Depressive Disorder - Alvarez-Mon et al., 2021 (15)

Appraisal Point	Positive/Methodologically Sound	Negative/Relatively Poor Methodology	Unknowns	Risk of Bias
Focus of the study	Investigates the role of regulatory T lymphocytes (Tregs) in MDD patients and their association with bacterial translocation.			Low
Methodological appropriateness	The cross-sectional design is appropriate for exploring immune dysregulation in MDD.		Lack of longitudinal data limits causal inferences.	Low
Sample recruitment	Homogeneous population (MDD vs. healthy controls), with exclusion criteria to limit confounding factors.		Details of recruitment methods are limited.	Low
Measurement accuracy	Tregs assessed using validated flow cytometry methods; biomarkers measured via ELISA.		Intra-assay variability not reported.	Low
Data collection adequacy	Comprehensive measurement of immune and gut biomarkers, including LBP, IL-10, and zonulin.		No assessment of other potential confounders like diet or lifestyle.	Low
Sample size sufficiency	Sufficient for exploratory analysis of associations.	Small sample size (30 MDD, 20 controls) may affect robustness.	Limited statistical power for subgroup analysis.	Medium

Presentation of results	Results clearly presented with medians, IQR, and Mann-Whitney U tests.		Lack of sensitivity analyses to confirm robustness.	Low
Data analysis rigor	Proper use of non-parametric tests and correlation analyses.		Interaction effects not explored.	Low
Clarity of findings	Findings support a connection between gut permeability and Treg alterations in MDD.		Mechanistic explanations remain speculative.	Low
Applicability to local population	Relevant for MDD patients with similar characteristics.	Single-center design limits broader generalizability.	Broader applicability to diverse populations unclear.	Medium/ Can't Tell
Research value	Advances understanding of immune and gut dysregulation in MDD.	Small sample size.Clinical implications require further exploration	.	Can't Tell

4) Biomarkers of intestinal permeability and blood-brain barrier permeability in adolescents with major depressive disorder - Wu et al., 2023 (16)

**Appraisal Point**	**Positive/Methodologically Sound**	**Negative/Relatively Poor Methodology**	**Unknowns**	**Risk of Bias**
Focus of the study	Investigates intestinal and blood-brain barrier permeability biomarkers in adolescent MDD.			Low
Methodological appropriateness	Cross-sectional design fits the study's exploratory objectives.		Limited in determining causality.	Low
Sample recruitment	Inclusion of drug-naïve MDD patients and matched controls.		Recruitment strategy for healthy controls not detailed.	Low
Measurement accuracy	Biomarkers measured using validated ELISA kits; samples handled appropriately.		Potential assay variability not discussed.	Low
Data collection adequacy	Comprehensive measurement of intestinal and blood-brain barrier biomarkers.		Limited adjustment for potential confounders like diet or stress.	Low
Sample size sufficiency	Sufficient for identifying associations but limited for subgroup analysis.	Relatively small sample size (50 MDD, 40 controls).		Medium
Presentation of results	Clear presentation of results with statistical rigor (ANOVA, MANCOVA, ROC analysis).		No detailed explanation for subgroup results inconsistencies.	Low
Data analysis rigor	Appropriate statistical tools used; confounders like age and BMI adjusted.		Additional confounders (e.g., socioeconomic factors) not considered.	Low
Clarity of findings	Findings support the role of permeability markers as potential biomarkers for MDD.		Biological mechanisms are speculative.	Low
Applicability to local population	Relevant to adolescents with similar demographic characteristics.	Generalizability limited by single-center design.	Broader applicability to diverse populations unclear.	Medium/ Can't Tell

Research value	Enhances understanding of gut-brain axis in adolescent MDD.	Relatively small sample size. Long-term clinical implications require further study.		Can't Tell

5) Iron status in Swiss adolescents with paediatric major depressive disorder and healthy controls: a matched case–control study - Osuna et al., 2024 (17)

**Appraisal Point**	**Positive/Methodologically Sound**	**Negative/Relatively Poor Methodology**	**Unknowns**	**Risk of Bias**
Focus of the study	Investigates iron status, systemic inflammation, and intestinal permeability in adolescents with pMDD.			Low
Methodological appropriateness	Case-control design suitable for comparing adolescents with pMDD to healthy controls.	.	Lack of longitudinal data prevents causal inference	Low
Sample recruitment	Well-matched groups (age, sex, education) and sufficient sample size (190 participants).		Response rates for some data (e.g., history of iron deficiency diagnosis) were lower in cases. Recruitment strategy for cases could be elaborated further.	Low
Measurement accuracy	Validated biomarkers (e.g., ELISA) for iron and inflammation; adjustment for inflammation using BRINDA.		Potential intra-assay variability not detailed.	Low
Data collection adequacy	Comprehensive assessments, including history of iron treatment and dietary habits.	.	Retrospective questionnaire responses may introduce recall bias	Low
Sample size sufficiency	Adequate to detect medium-to-large effect sizes as determined by power analysis.	Smaller subgroups (e.g., sex-specific comparisons) might reduce power.		Medium/ Can't Tell
Presentation of results	Results presented clearly (medians, IQR, adjusted values, multivariate analyses).		Lack of sensitivity analyses to explore robustness of key findings.	Low
Data analysis rigor	Multivariate logistic regression models adjusted for confounders (e.g., age, sex, BMI).		Potential confounders (e.g., socioeconomic factors) not fully addressed.	Low
Clarity of findings	Supports the inflammatory theory of depression; highlights differences in inflammatory markers and intestinal permeability.		Mechanistic explanations of findings remain speculative.	Low
Applicability to local population	Relevant for adolescents in similar healthcare and demographic settings.	Generalizability limited to Swiss adolescents (?)	Applicability to non-European populations unclear.	Medium/ Can't Tell
Research value	Adds to the understanding of the gut-brain axis and the role of iron in pMDD.			Low

6) Assessment of a multi-assay, serum-based biological diagnostic test for major depressive disorder: A Pilot and Replication Study - Papakostas et al., 2013 (19)

Appraisal Point	Positive/Methodologically Sound	Negative/Relatively Poor Methodology	Unknowns	Risk of Bias
Focus of the study	Evaluates the diagnostic performance of a serum-based test for MDD.			Low
Methodological appropriateness	Pilot and replication studies enhance reliability of findings.		Observational design does not establish causality.	Low
Sample recruitment	Inclusion of well-defined MDD and control groups; exclusion of potential confounders.		Recruitment strategy for controls is not elaborated in detail.	Low
Measurement accuracy	Biomarkers measured using validated ELISA techniques.		Intra-assay variability and reproducibility not discussed.	Low
Data collection adequacy	Comprehensive measurement of inflammatory, neurotrophic, and metabolic biomarkers.		Potential impact of lifestyle or environmental factors not considered.	Low
Sample size sufficiency	Adequate for primary analysis (power calculation included).	Relatively small sample size (n=36 and n=34 for MDD groups).	Limited for exploring subgroup or interaction effects.	Medium
Presentation of results	Results presented with statistical rigor (sensitivity, specificity, predictive values).		Limited discussion of inconsistencies across biomarkers.	Low
Data analysis rigor	Proper use of ANCOVA, logistic regression, and ROC analysis.		No sensitivity analysis performed.	Low
Clarity of findings	Supports the development of a composite biomarker-based diagnostic test for MDD.		Biological mechanisms underlying biomarker changes remain speculative.	Low
Applicability to local population	Relevant for clinical populations with similar demographics.	Limited generalizability due to single-country (USA) recruitment.	Broader applicability to diverse settings is unclear.	Medium
Research value	Provides promising evidence for a serum-based diagnostic test for MDD.	Relatively small sample size. Long-term utility in clinical practice needs further validation.		Medium/ Can't Tell

/) Association of markers of inflammation and intestinal permeability in suicidal patients with major mood disorders - Brouillet et al., 2023 (20)

Appraisal Point	Positive/Methodologically Sound	Negative/Relatively Poor Methodology	Unknowns	Risk of Bias
Focus of the study	Investigates inflammatory and intestinal permeability markers in suicidal patients with major mood disorders.			Low
Methodological appropriateness	Cross-sectional design is appropriate for studying associations between biomarkers and suicidality.		Causal relationships	Low

			cannot be determined.	
Sample recruitment	Inclusion criteria well-defined		No inclusion of healthy controls limits interpretability. Details on recruitment strategy not fully explained.	Low
Measurement accuracy	Biomarkers measured using validated ELISA and electrochemiluminescence assays.		Intra-assay variability and calibration data not detailed.	Low
Data collection adequacy	Comprehensive data collected on inflammatory and gut-related biomarkers.		Confounding variables such as diet and medications not fully controlled.	Low
Sample size sufficiency	Adequate for primary comparisons between groups. Large sample size (305 patients, including 137 BD and 168 MDD).	Missing data for some patients may affect subgroup analyses.		Medium/ Can't Tell
Presentation of results	Results clearly presented (means, SD, p-values, multivariate analysis).		No sensitivity analyses or external validation of findings.	Low
Data analysis rigor	Multivariate linear regressions adjusting for confounders like age, BMI, and smoking.		Further interactions (e.g., biomarkers x psychiatric diagnosis) not explored.	Low
Clarity of findings	Findings suggest CRP and IFABP as potential markers of suicidality.		Mechanistic pathways underlying biomarker associations remain unclear.	Low
Applicability to local population	Relevant for clinical populations with major mood disorders in psychiatric settings.	Single-center study limits broader applicability.	Generalizability to diverse populations is unclear.	Can't Tell
Research value	Contributes to understanding the gut-brain axis and its role in suicidality.		Translational relevance for interventions is speculative.	Low

8) Associations of Total, Cognitive/Affective, and Somatic Depressive Symptoms and Antidepressant Use with Cardiovascular Disease–Relevant Biomarkers in HIV: Veterans Aging Cohort Study - Stewart et al., 2020 (21)

**Appraisal Point**	**Positive/Methodologically Sound**	**Negative/Relatively Poor Methodology**	**Unknowns**	**Risk of Bias**
Focus of the study	Examines associations between depressive symptoms, antidepressant use, and CVD-relevant biomarkers in veterans with HIV.			Low
Methodological appropriateness	Observational cohort design suitable for evaluating associations in a large, diverse sample.		Cross-sectional analysis limits causal interpretations.	Low
Sample recruitment	Well-defined recruitment criteria	.	Recruitment strategy for	Low

			controls not detailed.	
Measurement accuracy	Biomarkers measured using validated assays with reported interassay variability.		No data on intra-assay variability or repeatability.	Low
Data collection adequacy	Comprehensive data on depression (PHQ-9), antidepressant use, and biomarkers (sCD14, IL-6, D-dimer).		Potential confounding factors, such as lifestyle variables, not fully controlled.	Low
Sample size sufficiency	Sufficient to detect moderate effect sizes; robust power calculation. Large sample size (1546 HIV-positive, 843 HIV-negative participants).		Small subgroups for antidepressant types may reduce statistical power.	Low
Presentation of results	Results clearly presented with appropriate statistical metrics		Lack of sensitivity analyses to confirm robustness of findings.	Low
Data analysis rigor	Use of multiple imputation and multivariable regression models.		Additional confounders (e.g., psychosocial factors) not explored.	Low
Clarity of findings	Clear associations identified between depressive symptoms and biomarkers, particularly for somatic symptoms.		Underlying mechanisms remain speculative.	Low
Applicability to local population	Relevant for HIV-positive veterans in clinical settings.	Generalizability to non-veteran populations and women is unclear.		Can't Tell
Research value	Adds to understanding of the interaction between depression, HIV, and CVD biomarkers.		Long-term clinical relevance needs further validation.	Low

9) Autoantibodies against the C-terminus of Lipopolysaccharide Binding Protein are elevated in young adults with psychiatric disease - Just et al., 2021 (22)

**Appraisal Point**	**Positive/Methodologically Sound**	**Negative/Relatively Poor Methodology**	**Unknowns**	**Risk of Bias**
Focus of the study	Investigates the role of autoantibodies against LBP in psychiatric disease and their potential immune function.			Low
Methodological appropriateness	Cross-sectional design suitable for exploring associations between biomarkers and psychiatric conditions.		Limited by the inability to establish causality.	Low
Sample recruitment	Large sample size (395 patients and 102 controls), diverse psychiatric diagnoses.		Recruitment strategy for controls not fully detailed.	Low
Measurement accuracy	Validated assays (e.g., ELISA, PEA) used for biomarkers; appropriate handling of samples.		Variability in biomarker reproducibility not extensively discussed.	Low

Data collection adequacy	Comprehensive measurement of inflammatory markers and LBP autoantibodies.		Limited adjustment for confounders like lifestyle factors or medications.	Low
Sample size sufficiency	Large sample size (395 patients and 102 controls), diverse psychiatric diagnoses. Sufficient to detect differences between groups; robust statistical analyses (e.g., Mann-Whitney, permutation tests).	Smaller subgroups may lack statistical power for certain analyses.		Low/Can't Tell
Presentation of results	Clear results with appropriate metrics (e.g., p-values, median fluorescence intensities).		Lack of sensitivity analyses or additional stratifications.	Low
Data analysis rigor	Multivariate analyses and appropriate non-parametric tests applied.		Interaction effects (e.g., between biomarkers and clinical outcomes) not explored.	Low
Clarity of findings	Findings suggest a novel role for LBP autoantibodies in psychiatric diseases.		Mechanistic pathways remain speculative.	Low
Applicability to local population	Relevant for young adults with psychiatric conditions in similar healthcare settings.	Generalizability limited by single-center design in Sweden.	Applicability to broader populations unclear.	Medium/ Can't Tell
Research value	Highlights potential biomarkers for psychiatric diseases and their immune modulation.		Long-term implications require further study.	Low

10) Elevated macrophage migration inhibitory factor and decreased transforming growth factor-beta levels in major depression — No influence of celecoxib treatment - Musil et al., 2011 (23)

**Appraisal Point**	**Positive/Methodologically Sound**	**Negative/Relatively Poor Methodology**	**Unknowns**	**Risk of Bias**
Focus of the study	Investigates immune markers (MIF, TGF-β, sCD14) in depressed patients treated with celecoxib vs. placebo.			Low
Methodological appropriateness	Randomized, double-blind, placebo-controlled trial with biomarker measurements.		Small sample size reduces power for subgroup analyses.	Low
Sample recruitment	Well-defined inclusion/exclusion criteria.	Limited generalizability due to single-center recruitment.	Recruitment strategy for controls not elaborated in detail.	Low
Measurement accuracy	Validated ELISA kits used for biomarker quantification; repeated measures over time.		Variability in biomarker reproducibility not extensively discussed.	Low
Data collection adequacy	Comprehensive data collection on immune parameters and clinical outcomes (HamD scores).		Confounding factors like diet or exercise not controlled.	Low
Sample size sufficiency	Adequate for initial exploratory analyses but underpowered for	High dropout rate reduces robustness of conclusions.		Medium

	complex interactions or subgroup analysis. 32 depressed patients and 20 healthy controls.			
Presentation of results	Clear presentation of results (means, SD, p-values, boxplot); significant changes identified over time.	.	No sensitivity analyses or validation of findings with external cohorts	Low
Data analysis rigor	Appropriate statistical tests (ANCOVA, regression models); adjustments for confounders (e.g., age, gender).		Lack of interaction analyses between clinical outcomes and biomarkers.	Low
Clarity of findings	Highlights immune dysregulation in MDD and potential roles of MIF and TGF-β.		Mechanistic insights remain speculative.	Low
Applicability to local population	Relevant for depressed patients with moderate-to-severe symptoms in clinical settings.	Generalizability limited by small and homogeneous sample.	Applicability to broader populations unclear.	Medium/ Can't Tell
Research value	Adds to understanding of immune mechanisms in MDD and potential therapeutic implications.		Long-term clinical relevance of findings remains uncertain.	Low

11) Evaluation of Serum Zonulin and Occludin Levels in Bipolar Disorder - Zengil & Laloğlu, 2023 (24)

**Appraisal Point**	**Positive/Methodologically Sound**	**Negative/Relatively Poor Methodology**	**Unknowns**	**Risk of Bias**
Focus of the study	Investigates serum zonulin and occludin as biomarkers in bipolar disorder.			Low
Methodological appropriateness	Cross-sectional design aligns with exploratory biomarker objectives.		Causal relationships cannot be determined.	Low
Sample recruitment	Includes well-matched patient and control groups (n = 44 per group).		Recruitment criteria for controls not detailed.	Low
Measurement accuracy	Biomarkers measured using validated ELISA kits with reported variability.		Potential batch effects not discussed.	Low
Data collection adequacy	Comprehensive measurement of inflammatory and gut permeability biomarkers.		No detailed control of confounding factors like diet and medication.	Low
Sample size sufficiency	Adequate for detecting significant differences between groups. Patient and control groups (n = 44 per group).	Small sample size limits robustness. Insufficient for subgroup or interaction analyses.		Medium
Presentation of results	Clear presentation with means, SD, and multivariate analysis results.		No sensitivity analysis to confirm findings.	Low
Data analysis rigor	Appropriate statistical methods (ANCOVA, ROC analysis, Tukey post hoc).		Interaction effects between biomarkers and clinical variables not explored.	Low

Clarity of findings	Demonstrates that zonulin and occludin levels are elevated in BD patients.		Mechanistic explanation remains speculative.	Low
Applicability to local population	Relevant for patients with bipolar disorder in clinical settings.	Generalizability limited due to single-center, Turkish population.	Broader applicability to diverse settings is unclear.	Medium/Can't Tell
Research value	Advances understanding of gut-brain axis in BD and its biomarker potential.		Clinical utility of findings requires further validation.	Can't Tell

12) Gut bacteriome and mood disorders in women with PCOS - Lee et al., 2024 (25)

Appraisal Point	Positive/Methodologically Sound	Negative/Relatively Poor Methodology	Unknowns	Risk of Bias
Focus of the study	Investigates the gut bacteriome's role in mood disorders (MDs) in women with PCOS.			Low
Methodological appropriateness	Population-based cohort study, appropriate for exploring associations.	.	Cross-sectional design limits causal inference	Low
Sample recruitment	Large, well-characterized cohort; age- and BMI-matched controls.		Recruitment strategy for controls less detailed.	Low
Measurement accuracy	Advanced 16S rRNA sequencing used for gut bacteriome profiling.		Variability in sequencing reproducibility not extensively discussed.	Low
Data collection adequacy	Comprehensive data on clinical features, mood disorders, and gut microbiota diversity.		Potential confounders like diet, medication use, and lifestyle factors not fully adjusted.	Low
Sample size sufficiency	Adequate for primary comparisons between groups. 102 women with PCOS (84 PCOS no-MD, 18 PCOS MD) and 205 BMI-matched women without PCOS (180 control no-MD, 25 control MD),	Small sample sizes in some subgroups (e.g., PCOS MD).	Limited power for subgroup	Medium
Presentation of results	Clear presentation with statistical rigor (e.g., alpha/beta diversity, FDR-corrected p-values).		No sensitivity analyses to confirm findings' robustness.	Low
Data analysis rigor	Multivariate methods (e.g., ANCOM-BC, Spearman correlations, PERMANOVA); appropriate statistical corrections (FDR).		Lack of longitudinal follow-up or temporal analyses.	Low
Clarity of findings	Identifies distinct gut bacteriome features associated with PCOS and MDs (e.g., reduced alpha diversity, lower Butyricicoccus abundance).		Mechanistic pathways remain speculative.	Low
Applicability to local population	Relevant for women with PCOS in clinical or research settings.	Single-center cohort from Finland may limit generalizability.	Broader applicability across diverse populations is unclear.	Medium/ Can't Tell
Research value	Adds valuable insights into the gut-brain axis in PCOS and its link to mood disorders.		Clinical implications and interventions	Low/ Can't Tell

			remain uncertain.	

13) In depression, bacterial translocation may drive inflammatory responses, oxidative and nitrosative stress (O&NS), and autoimmune responses directed against O&NS-damaged neoepitopes - Maes et al., 2013 (26)

**Appraisal Point**	**Positive/Methodologically Sound**	**Negative/Relatively Poor Methodology**	**Unknowns**	**Risk of Bias**
Focus of the study	Investigates associations between bacterial translocation and inflammatory/oxidative stress biomarkers in depression.			Low
Methodological appropriateness	Cross-sectional design aligns with exploratory objectives in biomarker research.		Causal relationships cannot be determined due to study design.	Low
Sample recruitment	well-defined inclusion/exclusion criteria.		Recruitment strategy for controls not fully detailed.	Low
Measurement accuracy	Biomarkers measured using validated ELISA and related assays; interassay variability reported (<6%).		Detailed calibration or reproducibility data not included.	Low
Data collection adequacy	Comprehensive biomarker data (e.g., IgM/IgA, cytokines, oxidative stress markers).	Small control group; homogeneity of socioeconomic status may limit generalizability.	Limited control for potential confounders (e.g., lifestyle, dietary factors).	Low
Sample size sufficiency	Includes 141 subjects (113 depressed patients, 28 controls); Adequate for primary analyses; exploratory multivariate and canonical analyses performed.	Limited power for subgroup analyses or less prevalent associations.		Medium
Presentation of results	Clear presentation of results using means, SD, p-values, and canonical correlations.	Lack of sensitivity analyses or external validation of findings.		Low
Data analysis rigor	Appropriate statistical tests (ANCOVA, regression, canonical correlation); adjustments for covariates like age and gender.		Interaction effects between biomarkers and clinical outcomes not fully explored.	Low
Clarity of findings	Highlights significant associations between bacterial translocation, O&NS, and autoimmune responses in depression.		Mechanistic explanations remain speculative.	Low
Applicability to local population	Relevant for clinical populations with similar socioeconomic backgrounds (Benelux countries).	Generalizability to other populations may be limited.		Medium
Research value	Provides novel insights into the gut-brain axis and its role in depression pathophysiology.		Clinical implications require further validation.	Low/ Can't Tell

14) Potential Biomarkers for Diagnosing Major Depressive Disorder Patients with Suicidal Ideation - Bai et al., 2021 (28)

**Appraisal Point**	**Positive/Methodologically Sound**	**Negative/Relatively Poor Methodology**	**Unknowns**	**Risk of Bias**
Focus of the study	Investigates potential serum biomarkers (AAT, HDLC,			Low

	APOA1, TRSF) for diagnosing MDD patients with suicidal ideation (SI).			
Methodological appropriateness	Cross-sectional design suitable for identifying diagnostic biomarkers.		Limited in establishing causal relationships between biomarkers and SI.	Low
Sample recruitment	Well-characterized groups: 86 HCs, 53 MDD with SI, 20 MDD without SI.	Small sample size for MDD without SI reduces robustness.	Recruitment strategy for controls less detailed.	Low
Measurement accuracy	Biomarkers measured with validated assays (e.g., ELISA); standardized handling of blood samples.		Reproducibility of assays not thoroughly reported.	Low
Data collection adequacy	Comprehensive biomarker data; multivariable adjustments for age, BMI, education, and depression scores.		Limited control for potential confounders like lifestyle factors.	Low
Sample size sufficiency	Adequate for primary analyses and biomarker identification. Well-characterized groups: 86 HCs, 53 MDD with SI, 20 MDD without SI.		Limited power for subgroup analyses (e.g., responders vs. non-responders).	Medium
Presentation of results	Clear presentation with AUC values, ROC curves, and multivariate analyses.	No sensitivity analysis for robustness of findings.		Low
Data analysis rigor	Multivariate methods (e.g., OPLS-DA, Random Forest); rigorous statistical corrections applied (VIP > 1.0, FDR).		Interaction effects between clinical outcomes and biomarkers not fully explored.	Low
Clarity of findings	Identifies a robust biomarker panel with high diagnostic accuracy for distinguishing MDD with SI from HCs and MDD without SI.		Mechanistic explanations of biomarker changes remain speculative	Low
Applicability to local population	Relevant for clinical populations in similar hospital settings.	Generalizability limited by single-center recruitment in Chongqing, China.	Applicability to other ethnic or demographic groups unclear.	Medium
Research value	Advances the understanding of biological markers in MDD with SI and their diagnostic potential.	Long-term implications require further validation. Small sample size for MDD without SI reduces robustness.		Medium

15) Lack of association of acute phase response proteins with hormone levels and antidepressant medication in perimenopausal depression - Karaoulanis et al., 2014 (30)

Appraisal Point	Positive/Methodologically Sound	Negative/Relatively Poor Methodology	Unknowns	Risk of Bias

Focus of the study	Investigates the relationship between acute-phase proteins, reproductive hormones, and SSRIs in perimenopausal depression.			Low
Methodological appropriateness	Cross-sectional design suitable for examining associations between acute-phase proteins and depression-related factors in perimenopausal women.		Cannot establish causal relationships or longitudinal effects.	Low
Sample recruitment	65 women recruited from outpatient clinics with clear inclusion/exclusion criteria; divided into depressed and non-depressed groups.		Representativeness of the control group is not fully addressed.	Low
Measurement accuracy	Acute-phase proteins measured using validated nephelometric methods; hormonal levels analyzed with standard lab techniques.		Lack of repeated measures to validate biomarker stability; potential variability in laboratory conditions not discussed.	Low
Data collection adequacy	Comprehensive collection of clinical data, depression severity (HAM-D 17), and biomarker levels; subgroup analysis based on SSRIs use.		Limited adjustment for potential confounders, such as diet, stress, or other medications.	Low
Sample size sufficiency	65 women recruited. Adequate for primary comparisons between depressed and non-depressed groups.	Insufficient for detailed subgroup analyses or exploring complex interactions between acute-phase proteins and hormones.		Medium
Presentation of results	Results presented with clear statistical analyses (e.g., Mann-Whitney, ANOVA); includes comparisons between groups and subgroups.		Lack of sensitivity analyses to confirm robustness of findings.	Low
Data analysis rigor	Explores correlations between acute-phase proteins and reproductive hormones; analyzes effects of SSRIs on biomarker levels.		Does not deeply explore interaction effects between biomarkers or hormonal fluctuations.	Low
Clarity of findings	Reports lack of significant differences in acute-phase proteins between depressed and non-depressed groups; highlights correlation between haptoglobin and estradiol in non-SSRI users.		Mechanistic explanations for findings remain speculative; limited discussion on implications for treatment or prevention strategies.	Low
Applicability to local population	Relevant for perimenopausal women with similar demographics; addresses hormonal and inflammatory factors in depression.	Limited generalizability to populations with different hormonal profiles, treatment practices, or healthcare access.	Limited to a single hospital in Greece; potential geographic and demographic selection bias.	Medium
Research value	Highlights complexity of interactions between acute-phase proteins, hormones, and SSRIs; contributes to understanding of perimenopausal depression pathophysiology.	Requires validation in larger, more diverse cohorts; findings do not support inflammatory hypotheses of depression in this population.		Can’t Tell

16) Sex differences in zonulin in affective disorders and associations with current mood symptoms - Maget et al., 2021 (31)

**Appraisal Point**	**Positive/Methodologically Sound**	**Negative/Relatively Poor Methodology**	**Unknowns**	**Risk of Bias**
Focus of the study	Investigates zonulin levels in euthymic vs. depressed individuals with affective disorders, exploring sex differences and associations with medication and symptoms.			Low
Methodological appropriateness	Cross-sectional design appropriate for exploring biomarker associations.		Limited ability to determine causality between zonulin and affective states.	Low
Sample recruitment	Clear inclusion/exclusion criteria.			Low
Measurement accuracy	Zonulin measured using validated ELISA assay with clear protocols.		Reproducibility and intra-assay variability not reported.	Low
Data collection adequacy	Comprehensive clinical and demographic data collected alongside biomarker measurements.		Potential confounding factors (e.g., diet, lifestyle) not fully controlled.	Low
Sample size sufficiency	121 participants (55 depressed, 37 euthymic). Adequate for group comparisons (euthymic vs. depressed) and sex differences.	Overrepresentation of male participants; lack of healthy controls limits interpretability.	Small subgroups reduce statistical power for secondary analyses.	Medium
Presentation of results	Clear presentation using medians (IQR), p-values, and subgroup analyses; no significant zonulin differences observed between mood states.	Lack of sensitivity analyses to confirm robustness.		Low
Data analysis rigor	Appropriate statistical tests (Mann-Whitney U, Spearman correlations); adjustments for covariates like BMI, age.		Interactions between clinical outcomes and zonulin not deeply explored.	Low
Clarity of findings	Reports no correlation between zonulin and mood state but highlights higher zonulin levels in women.		Mechanistic explanations remain speculative.	Low
Applicability to local population	Relevant to psychiatric patients in clinical settings similar to Graz, Austria.	Limited generalizability due to single-center recruitment.	Broader applicability to diverse populations unclear.	Medium
Research value	Advances understanding of intestinal permeability in affective disorders and highlights sex differences in zonulin.	lack of healthy controls limits interpretability.	Long-term implications require further validation.	Can’t tell

17) The gut-brain barrier in major depression: Intestinal mucosal dysfunction with an increased translocation of LPS from gram-negative enterobacteria (leaky gut) plays a role in the inflammatory pathophysiology of depression - Maes et al., 2008 (33)

**Appraisal Point**	**Positive/Methodologically Sound**	**Negative/Relatively Poor Methodology**	**Unknowns**	**Risk of Bias**
Focus of the study	Explores the role of intestinal mucosal dysfunction and increased LPS translocation in the			Low

	inflammatory pathophysiology of major depressive disorder (MDD).			
Methodological appropriateness	Cross-sectional design suitable for evaluating the association between gut barrier dysfunction and inflammatory responses in MDD.		Lacks a longitudinal component to establish causality between leaky gut and depressive symptoms.	Low
Sample recruitment	Clear inclusion/exclusion criteria.		Limited to a single outpatient clinic in Belgium; potential selection bias from recruiting staff and family members as controls.	Cant'Tell
Measurement accuracy	Biomarkers (IgM and IgA against LPS of enterobacteria) measured using validated ELISA methods; interassay variability reported as <10%.		Does not include repeated measures for biomarker stability or additional validation of results.	Low
Data collection adequacy	Comprehensive collection of biomarkers, inflammatory markers, and depressive symptoms using validated scales.		Limited adjustment for potential confounders, such as dietary habits, lifestyle, or other comorbid conditions.	Low
Sample size sufficiency	Includes 51 participants (28 MDD patients and 23 healthy controls) Adequate for primary comparisons between MDD and control groups.	Insufficient for detailed subgroup or stratified analyses (e.g., by severity of depression or comorbid conditions).		Medium
Presentation of results	Results presented with robust statistical analyses (e.g., ANOVA, ROC curves); significant diagnostic performance (AUC = 90.8%).		Lack of sensitivity analyses to validate the robustness of the associations reported.	Low
Data analysis rigor	Explores relationships between biomarkers and symptom profiles using multivariate regression and canonical correlation analysis.		Interaction effects between biomarkers, inflammatory pathways, and depressive symptoms not deeply explored.	Low
Clarity of findings	Reports significant associations between increased IgM/IgA levels against LPS and symptoms of fatigue, autonomic dysfunction, and gastrointestinal issues in MDD patients.	Mechanistic explanations for findings remain speculative; limited discussion of potential interventions or treatments.		Low
Applicability to local population	Relevant for populations with similar clinical and demographic profiles; highlights gut-brain axis contributions to MDD.	Limited applicability to broader populations with different socio-economic, cultural, or dietary backgrounds.		Medium
Research value	Contributes to understanding of the gut-brain axis and its role in MDD; suggests diagnostic utility of gut permeability biomarkers.	.	Findings require validation in larger and more diverse cohorts; no direct implications for treatment or prevention strategies provided	Can't Tell

18) Upregulation of the nitrosylome in bipolar disorder type 1 (BP1) and major depression, but not BP2: Increased IgM antibodies to nitrosylated conjugates are associated with indicants of leaky gut - Maes et al., 2019 (34)

Appraisal Point	Positive/Methodologically Sound	Negative/Relatively Poor Methodology	Unknowns	Risk of Bias
Focus of the study	Examines the role of nitrosylation and bacterial translocation in the inflammatory pathophysiology of mood disorders, including bipolar disorder type 1 (BP1), type 2 (BP2), and major depression (MDD).			Low
Methodological appropriateness	Cross-sectional design suitable for exploring associations between oxidative stress, bacterial translocation, and mood disorders.		Lack of longitudinal data to confirm causal relationships between nitrosylation and mood disorder progression.	Low
Sample recruitment	Clear inclusion/exclusion criteria applied.		Controls were predominantly staff or relatives, potentially introducing selection bias.	Cant'Tell
Measurement accuracy	Biomarkers measured using validated ELISA methods with low inter-assay variability (<10%); robust methods for nitrosylation and bacterial translocation indices.		Limited discussion on assay reproducibility and potential variability across different labs or conditions.	Low
Data collection adequacy	Comprehensive collection of biomarkers (IgM responses to nitrosylated adducts, oxidative stress markers, LPS responses) alongside psychiatric evaluations (HAM-D).		Limited adjustment for potential confounders, such as diet, medication, or comorbid conditions.	Low
Sample size sufficiency	Includes 118 participants (22 healthy controls, 27 BP1, 25 BP2, and 44 MDD patients). Adequate for primary comparisons between diagnostic groups.	Insufficient for subgroup analyses or detailed exploration of interactions between variables.		Medium
Presentation of results	Results presented with appropriate statistical analyses (e.g., ANOVA, logistic regression, correlations); significant associations clearly described.		Lack of sensitivity analyses to test the robustness of findings.	Low
Data analysis rigor	Explores complex relationships between nitrosylation, oxidative stress, and bacterial translocation using multivariate and correlation analyses.		Does not fully explore the interaction effects of biomarkers across different diagnostic groups.	Low
Clarity of findings	Identifies significant nitrosylation in BP1 and MDD patients compared to BP2 and controls; shows associations between bacterial translocation, oxidative stress, and mood disorders.		Mechanistic explanations for differences between BP1 and BP2 remain speculative.	Low
Applicability to local population	Relevant for populations with similar clinical and demographic profiles; highlights the role of the gut-brain axis in mood disorders.	Limited applicability to broader populations with different socio-economic, cultural, or healthcare contexts.		Medium
Research value	Advances understanding of the gut-brain axis in mood disorders and introduces biomarkers (IgM to NO-	.	Requires validation in larger, diverse cohorts; no direct implications for clinical practice or	Cant'Tell

	adducts) as potential diagnostic tools.		intervention strategies provided	

19) Increased human intestinal barrier permeability plasma biomarkers zonulin and FABP2 correlated with plasma LPS and altered gut microbiome in anxiety or depression" (Stevens et al., 2018)

Appraisal Point	Positive/Methodologically Sound	Negative/Relatively Poor Methodology	Unknowns	Risk of Bias
Focus of the study	Clearly examines the relationship between gut permeability and depression.			Low
Methodological appropriateness	Cross-sectional design fits the aim to explore associations.		Does not allow causal inference.	Low
Sample recruitment	Clear inclusion/exclusion criteria.		Potential variability in biomarker processing not explicitly detailed.	Low
Measurement accuracy	Biomarkers measured using validated ELISA kits.		No mention of inter-assay variability or blinding of laboratory assessments.	Low
Data collection adequacy	Clinical and demographic data collected.		No adjustment for potential confounders like diet, smoking, or physical activity.	Low
Sample size sufficiency		Small sample (n=50); insufficient for complex subgroup analyses.		Medium
Presentation of results	Results clearly presented with tables and statistical parameters (mean ± SD, p-value, ANOVA, Boxplot).		No sensitivity analysis	Low
Data analysis rigor	Basic statistical tests reported (group comparisons, correlation).			Low
Clarity of findings	Findings aligned with gut-brain axis hypotheses.		Mechanistic interpretation speculative.	Low
Applicability to local population	Results relevant to similar Western populations.	Limited by single-site design; generalizability unclear.	Applicability outside Sweden not discussed	Medium
Research value	Provides preliminary evidence for gut permeability markers in MDD.	Clinical utility not evaluated; no longitudinal outcomes.		Can't Tell

20) Elevated plasma intestinal fatty acid binding protein and aberrant lipid metabolism predict post-stroke depression - Zhong et al., 2022 (35)

Appraisal Point	Positive/Methodologically Sound	Negative/Relatively Poor Methodology	Unknowns	Risk of Bias
Focus of the study	Investigates the association between plasma intestinal fatty acid binding protein (iFABP), lipid metabolism, and post-stroke depression (PSD).			Low

Methodological appropriateness	Prospective design suitable for identifying potential biomarkers for PSD.		Lack of direct experimental validation for mechanistic pathways.	Low
Sample recruitment	144 participants divided into three groups (48 PSD patients, 48 stroke without depression, and 48 healthy controls), with clear inclusion/exclusion criteria.		Recruitment limited to a single hospital in China, possibly affecting generalizability.	Low
Measurement accuracy	Biomarkers (e.g., iFABP, ApoA1, HDL-C) measured using validated ELISA methods with clear protocols.		Lack of information on inter-laboratory reproducibility of assays.	Low
Data collection adequacy	Comprehensive assessment of biomarkers, lipid metabolism, and demographic data; use of validated scales (HAMD, HAMA) for depression severity.	.	Limited control for potential confounders such as diet, medication, and comorbid conditions	Low
Sample size sufficiency	Adequate for primary comparisons between PSD and non-PSD groups, as well as healthy controls.	Insufficient for subgroup analyses or detecting complex interactions between multiple biomarkers.		Medium
Presentation of results	Results presented with clear statistical analyses (e.g., OPLS model, ROC analysis); effective differentiation between PSD and non-PSD groups.		Lack of sensitivity analyses to confirm robustness of findings.	Low
Data analysis rigor	Multivariate analysis used to identify potential biomarkers; correlation analysis performed to explore relationships with depression scores.		Interaction effects between iFABP and lipid metabolism not fully explored; longitudinal validation missing.	Low
Clarity of findings	Identifies iFABP, ApoA1, HDL-C, and Lp(a) as potential PSD biomarkers; reports significant correlations with depression severity scores.		Mechanistic explanations for biomarker interactions remain speculative.	Low
Applicability to local population	Relevant for clinical populations with similar demographics; findings align with the "gut-brain axis" hypothesis in PSD.	Limited applicability to diverse populations outside the study region.	Generalizability to other healthcare settings remains uncertain.	Medium
Research value	Advances understanding of the gut-brain axis and lipid metabolism in PSD; highlights potential biomarkers for early diagnosis and treatment monitoring.	Requires validation in larger, multi-center studies before clinical implementation.		Can't Tell

21) An exploratory analysis on the association between suicidal ideation and the microbiome in patients with or without major depressive disorder" - Chen and Wu, 2024(36) (36) (This is a preprint work that has not yet been peer-reviewed).

**Appraisal Point**	**Positive/Methodologically Sound**	**Negative/Relatively Poor Methodology**	**Unknowns**	**Risk of Bias**
Focus of the study	Examines the diversity and abundance of gut microbiota in patients with or without suicidal ideation and major depressive disorder (MDD).			Low

Methodological appropriateness	Cross-sectional design suitable for exploring associations between microbiota and suicidal ideation.		Cannot establish causal relationships.	Low
Sample recruitment	140 participants recruited from psychiatric outpatient clinics and as healthy controls. Clear inclusion/exclusion criteria were applied.		Recruitment limited to a single hospital in Taiwan; potential geographical and socio-economic selection bias.	Low
Measurement accuracy	Microbiota biomarkers analyzed using validated 16S rRNA methods; accurate measurement of intestinal permeability and DHEA markers.		Potential influence of unmeasured confounders (e.g., diet, lifestyle).	Low
Data collection adequacy	Data collected using validated questionnaires (e.g., BSSI, PHQ-9); biological markers and microbiomes analyzed using robust approaches (e.g., multivariate regression).		Limited adjustment for confounders such as dietary and environmental factors. Details on fecal sample collection methods are not specified.	Low
Sample size sufficiency	140 participants recruited from psychiatric outpatient clinics and as healthy controls. Adequate for primary analyses among the three groups (MDD with suicidal ideation, MDD without suicidal ideation, and healthy controls).	Limited power for subgroup analyses or exploring complex interactions.		Medium
Presentation of results	Results clearly presented with appropriate statistical analyses (e.g., multivariate linear regression, alpha and beta diversity analysis).		Lack of sensitivity analyses to validate the robustness of reported associations.	Low
Data analysis rigor	Use of multivariate regression models to control for demographic and psychometric variables; separate analysis for ideation and intensity of suicidal thoughts.		Complex interactions between microbiota, psychological variables, and biomarkers not fully explored.	Low
Clarity of findings	Reports significant associations between Phascolarctobacterium and suicidal ideation; clear distinction between alpha and beta diversity among study groups.		Lack of a definitive mechanistic explanation for the observed relationships.	Low
Applicability to local population	Relevant for clinical populations similar to the study cohort (Taiwan); contributes to understanding of microbiota in relation to mental health.	Limited applicability to contexts with different cultural, environmental, or demographic characteristics.	Generalizability beyond Taiwan remains uncertain.	Can't Tell
Research value	Advances understanding of the gut-brain axis in depression and suicide risk; identifies potential therapeutic targets.	Insufficient evidence for immediate clinical implications.	Requires confirmation in longitudinal studies?	Can't Tell

22) Prediction of Post-Stroke Depression with Combined Blood Biomarkers IL-6, TNF-a, and Fatty Acid Binding Protein: A Prospective Study - Wang et al., 2023 (37)

**Appraisal Point**	**Positive/Methodologically Sound**	**Negative/Relatively Poor Methodology**	**Unknowns**	**Risk of Bias**
Focus of the study	Examines the predictive value of IL-6, TNF-a, and iFABP for post-			Low

	stroke depression (PSD) occurrence.			
Methodological appropriateness	Prospective design suitable for studying the association between biomarkers and PSD.		Limited by lack of experimental validation for causal pathways.	Low
Sample recruitment	91 PSD patients and 208 non-PSD stroke patients recruited with well-defined inclusion/exclusion criteria.		Recruitment from a single hospital in China may limit generalizability.	Low
Measurement accuracy	Biomarkers measured using validated ELISA methods with appropriate controls.		Specific reproducibility and inter-laboratory reliability of assays not fully discussed.	Low
Data collection adequacy	Comprehensive collection of clinical and demographic data, alongside biomarker analysis.	.	Limited control for potential confounders such as diet, medications, and comorbid conditions	Low
Sample size sufficiency	Adequate for primary comparisons between PSD and non-PSD groups.	Insufficient for subgroup analyses or detecting complex interactions between biomarkers.		Medium
Presentation of results	Results presented with robust statistical analyses (e.g., logistic regression, ROC analysis); clear separation of PSD and non-PSD groups demonstrated.	Lack of sensitivity analyses to test the robustness of findings.		Low
Data analysis rigor	Logistic regression and ROC analysis used to explore predictive value of combined biomarkers; correlation analysis with clinical scales performed.	Interaction effects between biomarkers not deeply explored; absence of longitudinal validation.		Low
Clarity of findings	Clearly reports significant associations between combined biomarkers and PSD; highlights the superior predictive value of combined biomarkers over individual markers.	Mechanistic explanations for biomarker interactions and their role in PSD development remain speculative.		Low
Applicability to local population	Relevant for clinical populations with similar stroke demographics; supports hypothesis of intestinal permeability and inflammation in PSD.	Limited generalizability due to specific socio-economic and cultural context of study cohort.	Applicability to other populations (e.g., Western settings) unclear.	Can't Tell
Research value	Provides evidence for the gut-brain axis hypothesis in PSD; identifies biomarkers with potential for clinical screening.	Further validation in larger, more diverse cohorts is necessary for clinical application.		Low/ Can't Tell

Appendix S4 – Quality of evidence synthesis

Table S2. Quality of evidence of studies included in the quantitative analysis

Study; Year	*1) Did the study address a clearly focused issue?*	*2) Did the authors use an appropriate method to answer their question?*	*3) Were the subjects recruited in an acceptable way?*	*4) Were the measures accurately measured to reduce bias?*	*5) Were the data collected in a way that addressed the research issue?*	*6) Did the study have eNough participants to minimise the play of chance?*	*7) How are the results presented and what is the main result?*	*8) Was the data analysis sufficiently rigorous?*	*9) Is there a clear statement of findings?*	*10) Can the results be applied to the local population*	*11) How valuable is the research?*	*Total (n/11)*
Ohlsson et al., 2019	Yes	Yes	Yes	Yes	Yes	No	Median (IQR); p-values; ANOVA Boxplots	Yes	Yes	Can't Tell	Can't Tell	8
Alvarez-Mon et al., 2019	Yes	Yes	Yes	Yes	Yes	No	Mean ± SD; Median (IQR)	Yes	Yes	Can't Tell	Can't Tell	8
Alvarez-Mon et al., 2021	Yes	Yes	Yes	Yes	Yes	No	Median (IQR); Absolute Counts; p-values; Boxplots	Yes	Yes	Can't Tell	Can't Tell	8
Wu et al., 2023	Yes	Yes	Yes	Yes	Yes	No	Mean ± SD; Median (IQR); ROC curve	Yes	Yes	Can't Tell	Can't Tell	8
Osuna et al., 2024	Yes	Yes	Yes	Yes	Yes	Can't Tell	Mean ± SD; Median (IQR); p-values;	Yes	Yes	Can't Tell	Yes	9
Papakostas et al., 2013	Yes	Yes	Yes	Yes	Yes	No	Mean ± SD; p-values; ROC curve	Yes	Yes	No	Can't Tell	8
Brouillet et al., 2023	Yes	Yes	Yes	Yes	Yes	Can't Tell	Mean ± SD; p-values, multivariate regression analysis	Yes	Yes	Can't Tell	Yes	9
Stewart et al., 2020	Yes	Yes	Yes	Yes	Yes	Yes	Median (IQR); mean ± SD; p-values; Multivariate regression analysis	Yes	Yes	Can't Tell	Yes	10

Just et al., 2021	Yes	Yes	Yes	Yes	Yes	Can’t Tell	Median (IQR); Mean ± SD; p-values,	Yes	Yes	Can’t Tell	Yes	9
Musil et al., 2011	Yes	Yes	Yes	Yes	Yes	No	Mean ± SD; p-values, Linear regression models; Boxplot	Yes	Yes	Can’t Tell	Yes	9
Zengil & Laloğlu, 2023	Yes	Yes	Yes	Yes	Yes	No	Mean ± SD; p-values, Linear regression models; ANCOVA; ROC curves	Yes	Yes	Can’t Tell	Can’t Tell	8
Lee et al., 2024	Yes	Yes	Yes	Yes	Yes	No	Median (IQR); p-values; Boxplot; PERMANOVA	Yes	Yes	Can’t Tell	Can’t Tell	8
Maes et al., 2013	Yes	Yes	Yes	Yes	Yes	No	Mean ± SD; p-values, correlation analysis	Yes	Yes	No	Can’t Tell	8
Bai et al, 2021	Yes	Yes	Yes	Yes	Yes	No	Median (IQR); p-values; Multivariate analysis; correlation analysis; ROC curves	Yes	Yes	No	No	8
Karaoulanis et al., 2014	Yes	Yes	Yes	Yes	Yes	No	Mean ± SD; Median (IQR); p-values; Linear regression analysis	Yes	Yes	No	Can’t Tell	8
Maget et al., 2021	Yes	Yes	Yes	Yes	Yes	No	Mean ± SD; p-values; Spearman's rank correlation coefficient	Yes	Yes	No	Can’t Tell	8
Maes et al., 2008	Yes	Yes	Can’t Tell	Yes	Yes	No	Mean ± SD; p-values; ROC curve	Yes	Yes	No	Can’t Tell	7

Maes et al., 2019	Yes	Yes	Can't Tell	Yes	Yes	No	Mean ± SD; p-values; Multivariate analysis (GLM)	Yes	Yes	No	Can't Tell	7
Stevens et al., 2018	Yes	Yes	Yes	Yes	Yes	No	Median (IQR); p-values; ANOVA; Boxplots	Yes	Yes	No	Can't Tell	8
Zhong et al., 2022	Yes	Yes	Yes	Yes	Yes	No	Medians (IQR); p-values,;ANOVA	Yes	Yes	No	Can't Tell	8
Chen and Wu, 2024; (Preprint)	Yes	Yes	Yes	Yes	Yes	No	Correlation coefficients, regression models	Yes	Yes	Can't Tell	Can't Tell	8
Wang et al, 2023	Yes	Yes	Yes	Yes	Yes	Can't Tell	t-tests, Logistic regression, ROC curves	Yes	Yes	Can't Tell	Can't Tell	8

Appendix S5

Quantitative analyses for primary outcomes: depressive symptoms

LPS

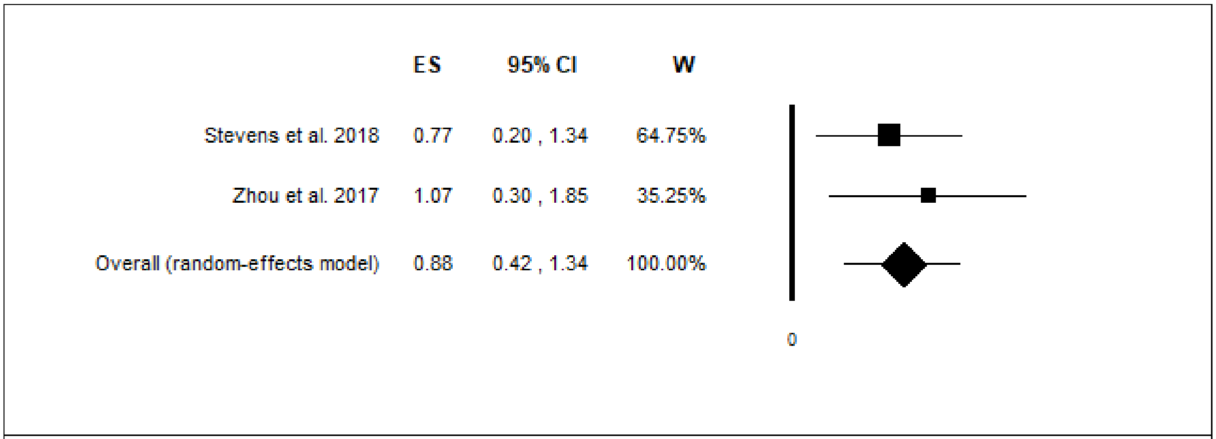

Overall effect ($p<0.001$), N=78, n1=36, n2=42

Heterogeneity ($Q(1)=0.39$, $p=0.532$). $I^2=0.00$; $T^2=0.00$; $T=0.00$

Sensitivity: The analysis of standardized residuals did not reveal any outlier studies.

Quantitative analyses for primary outcomes: suicidal risk

Zonulin

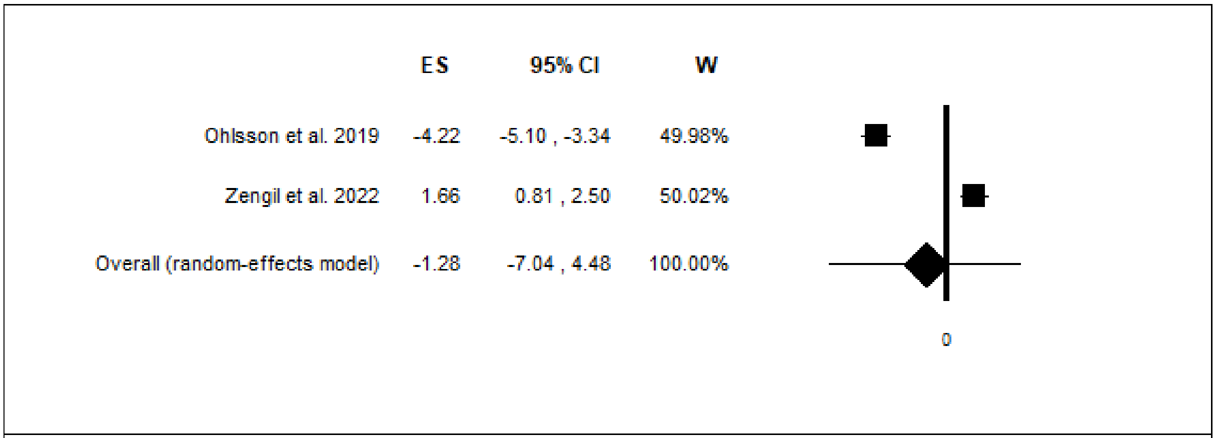

Overall effect ($p=0.663$), N=122, n1=61, n2=61

Heterogeneity ($Q(7)=88.91$, $p<0.001$). $I^2=98.88$; $T^2=17.08$; $T=4.13$

Sensitivity: NA

LBP

	ES	95% CI	W
Brouillet et al. 2024 (female)	0.18	-0.10 , 0.46	66.92%
Brouillet et al. 2024 (male)	0.25	-0.14 , 0.65	33.08%
Overall (random-effects model)	0.20	-0.02 , 0.43	100.00%

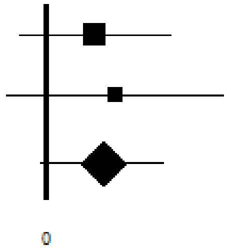

Overall effect ($p=0.078$), N=305, n1=133, n2=172

Heterogeneity ($Q(1)=0.09$, $p<0.762$). $I^2=0.00$; $T^2=0.00$; $T=0.00$

Sensitivity: NA

LPS

	ES	95% CI	W
Brouillet et al. 2024 (female)	-0.31	-0.59 , -0.03	64.96%
Brouillet et al. 2024 (male)	-0.58	-0.98 , -0.18	35.04%
Overall (random-effects model)	-0.40	-0.65 , -0.15	100.00%

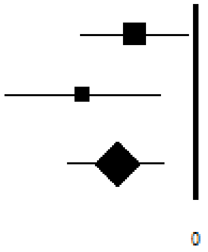

Overall effect ($p=0.002$), N=305, n1=133, n2=172

Heterogeneity ($Q(1)=1.17$, $p=0.280$). $I^2=14.24$; $T^2=0.01$; $T=0.07$

Sensitivity: NA

sCD14

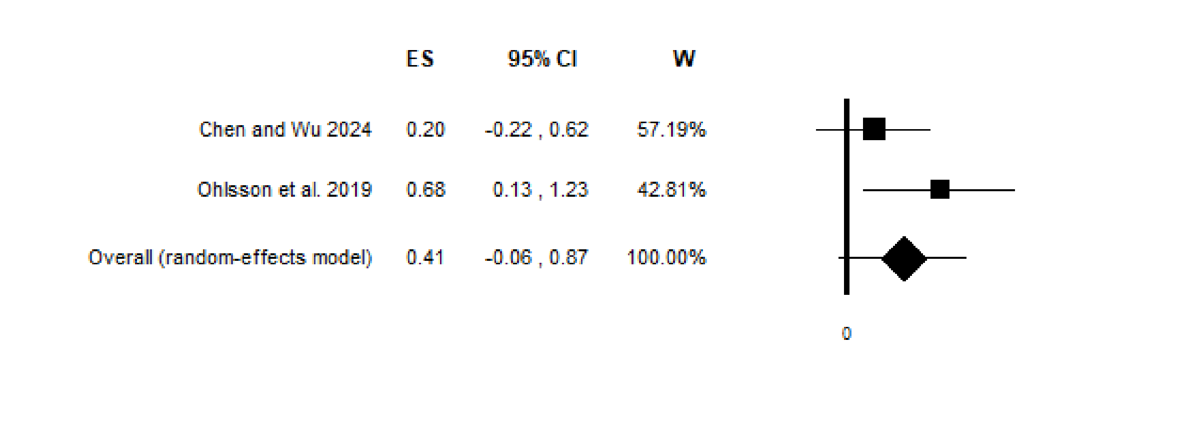

Overall effect (p=0.087), N=157, n1=98, n2=59

Heterogeneity (Q(1)=1.84, p=0.175). I^2=45.55; T^2=0.05; T=0.23

Sensitivity: NA

Appendix S6

Publication bias analyses for primary outcomes: depressive symptoms

I-FABP

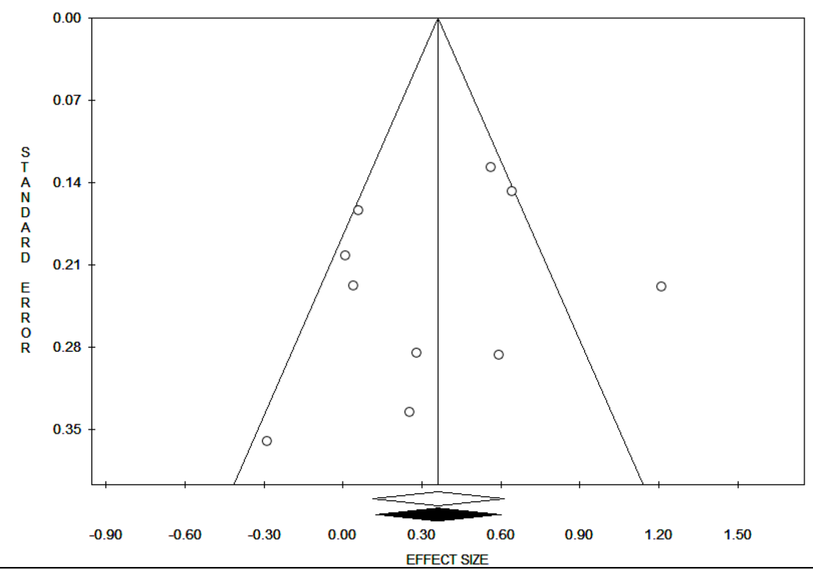

Egger's linear regression test Sig.=0.453
Begg and Mazumdar's rank correlation test Sig.=0.788
Fail Safe N.**71** (Rosenthal's rule of thumb 5k+10=60)

Zonulin

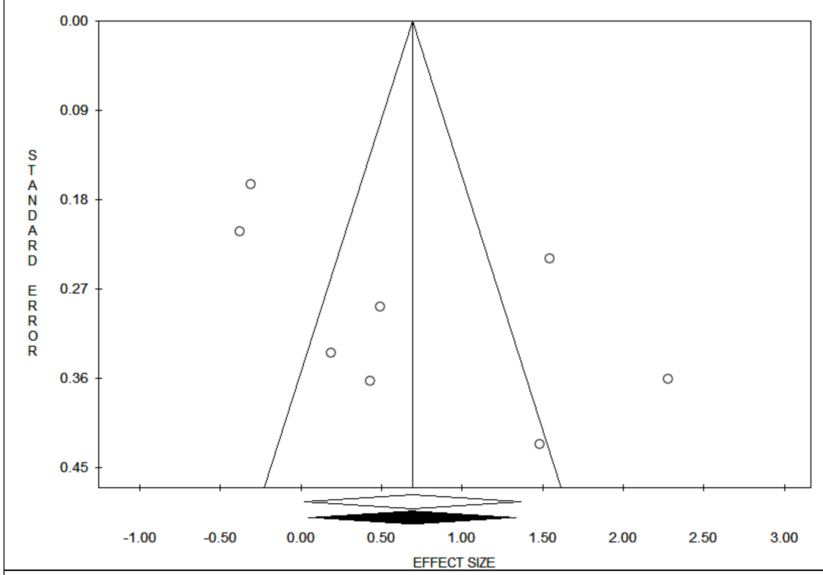

Egger's linear regression test Sig.=0.085
Begg and Mazumdar's rank correlation test Sig.=0.138
Fail Safe N.**59** (Rosenthal's rule of thumb 5k+10=50)

LBP

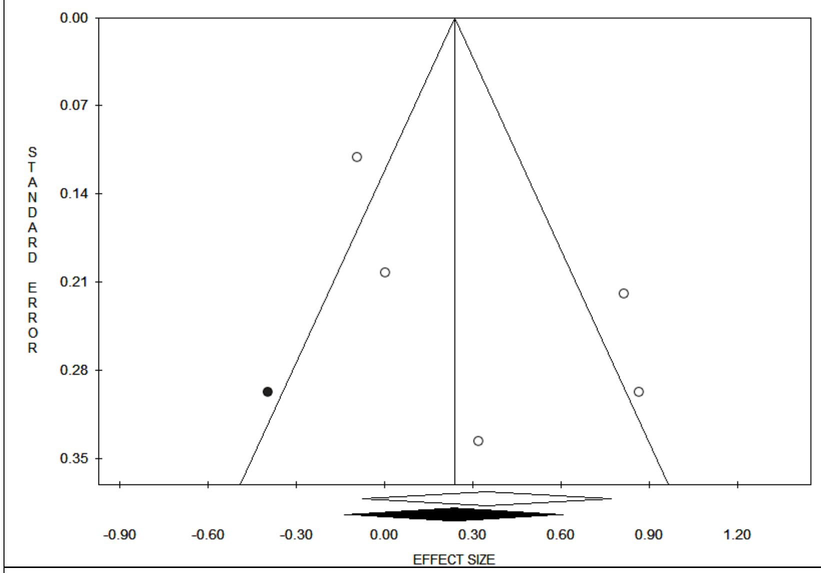

Egger’s linear regression test Sig.=0.147
Begg and Mazumdar’s rank correlation test Sig.=0.327
Fail Safe N.7 (Rosenthal’s rule of thumb 5k+10=35)

LPS: NA

Antibodies to endotoxins

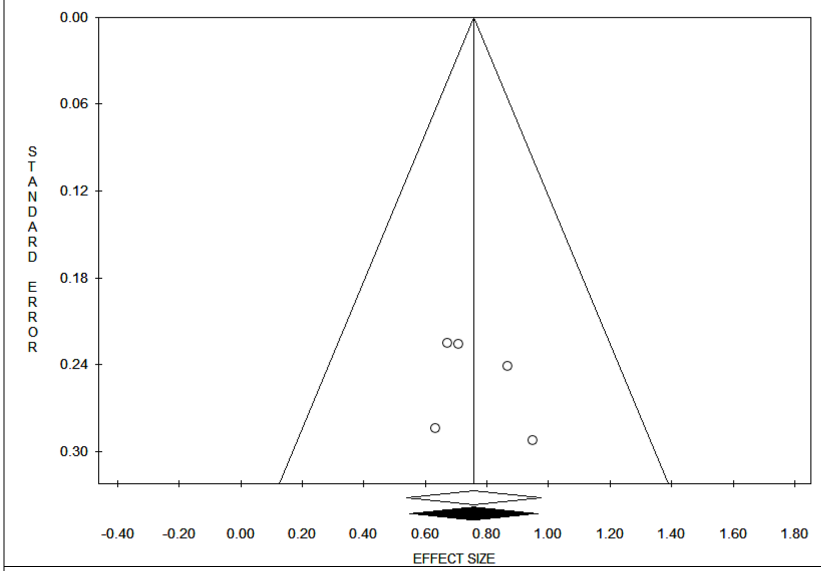

Egger’s linear regression test Sig.=0.534
Begg and Mazumdar’s rank correlation test Sig.=0.327
Fail Safe N.**55** (Rosenthal’s rule of thumb 5k+10=35)

A-1-AT

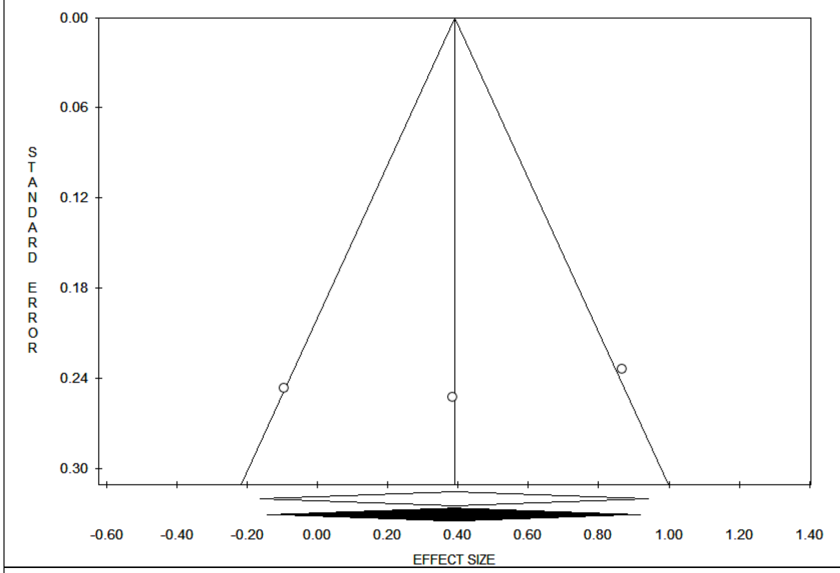

Egger's linear regression test Sig.=0.514
Begg and Mazumdar's rank correlation test Sig.=0.602
Fail Safe N.3 (Rosenthal's rule of thumb 5k+10=25)

sCD14

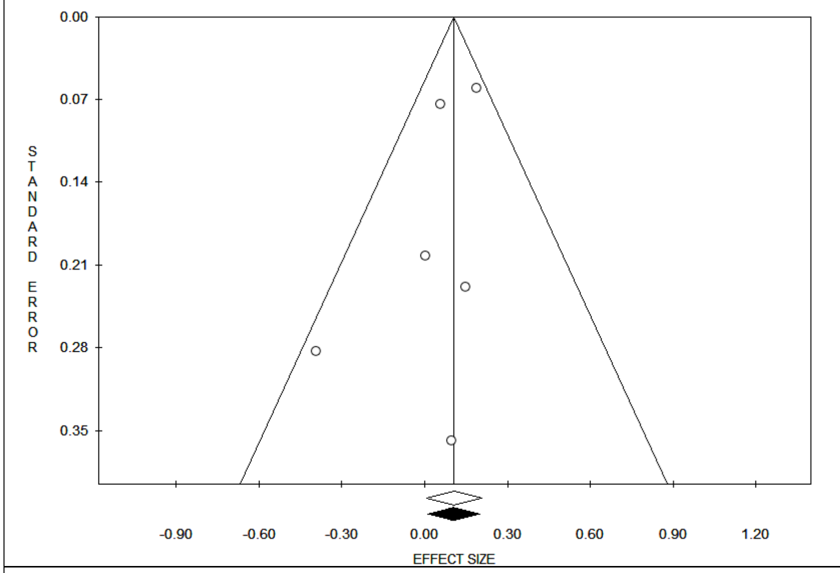

Egger's linear regression test Sig.=0.238
Begg and Mazumdar's rank correlation test Sig.=0.573
Fail Safe N.0 (Rosenthal's rule of thumb 5k+10=40)

Appendix S7

Moderator analysis for depressive symptoms severity

Zonulin

	ES	95% CI	W	N	N1	N2
Alvarez-Mon et al. 2019	0.19	-0.47 / 0.85	42.57%	36	22	14
Alvarez-Mon et al. 2021	0.50	-0.07 / 1.06	57.43%	50	30	20
Mild	0.37	-0.06 / 0.79	100.00%	86	52	34
Maget et al. 2021	-0.38	-0.79 / 0.04	100.00%	92	55	37
Mild to Moderate	-0.38	-0.79 / 0.04	100.00%	92	55	37
Ohlsson et al. 2019	0.43	-0.28 / 1.14	47.01%	30	13	17
Wu et al. 2023	1.55	1.08 / 2.02	52.99%	90	50	40
Moderate to Severe	1.02	-0.07 / 2.11	100.00%	120	63	57
Lee et al. 2023	-0.31	-0.64 / 0.01	34.52%	305	43	262
Zengil and Laloğlu 2022	1.49	0.65 / 2.32	32.40%	51	7	44
Stevens et al. 2018	2.28	1.57 / 2.99	33.08%	50	22	28
Unclear	1.13	-0.65 / 2.90	100.00%	406	72	334

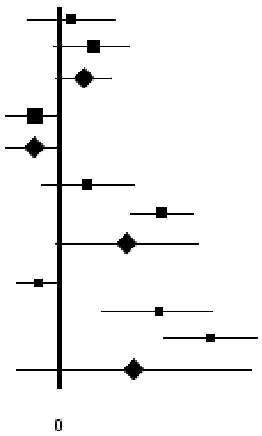

	Q	df	Sig.	I^2	T^2
Mild	0.48	1	0.489	0.00	0.00
Moderate to Severe	6.55	1	0.011	84.72	0.52
Unclear	51.53	2	0.000	96.12	2.35

LBP

	ES	95% CI	W	N	N1	N2
Zhong et al. 2022	0.00	-0.39 , 0.40	39.81%	96	48	48
Alvarez-Mon et al. 2019	0.32	-0.34 , 0.98	28.54%	36	22	14
Alvarez-Mon et al. 2021	0.86	0.28 , 1.45	31.64%	50	30	20
Mild	0.37	-0.16 , 0.89	100.00%	182	100	82
Just et al. 2021	-0.09	-0.31 , 0.12	100.00%	497	395	102
Mild to Moderate	-0.09	-0.31 , 0.12	100.00%	497	395	102
Wu et al. 2023	0.81	0.38 , 1.24	100.00%	90	50	40
Moderate to Severe	0.81	0.38 , 1.24	100.00%	90	50	40

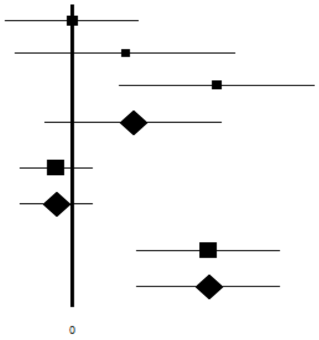

	Q	df	Sig.	I^2	T^2
Mild	5.77	2	0.056	65.32	0.14

LPS : NA

sCD14

	ES	95% CI	W	Sig.	N	N1	N2
Zhong et al. 2022	0.00	-0.39 / 0.40	56.05%	0.990	96	48	48
Chen and Wu 2024	0.15	-0.30 / 0.60	43.95%	0.520	76	34	42
Mild	0.07	-0.23 / 0.36	100.00%	0.663	172	82	90
Ohlsson et al. 2019	0.10	-0.61 / 0.80	100.00%	0.786	30	13	17
Moderate to Severe	0.10	-0.61 / 0.80	100.00%	0.786	30	13	17
Musil et al. 2011	-0.39	-0.95 / 0.16	100.00%	0.168	52	32	20
Severe	-0.39	-0.95 / 0.16	100.00%	0.168	52	32	20

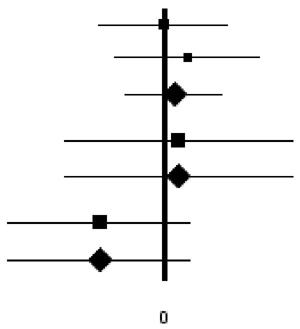

	Q	df	Sig.	I^2	T^2
Mild	0.22	1	0.636	0.00	0.00

Ab to LPS

	ES	95% CI	W	Sig.	N	N1	N2
Maes et al. 2019 (IgM/IgA)	0.87	0.39 / 1.34	100.00%	0.000	118	96	22
Severe	0.87	0.39 / 1.34	100.00%	0.000	118	96	22
Maes et al. 2008 (IgM)	0.63	0.08 / 1.19	19.57%	0.026	51	28	23
Maes et al. 2012 (IgM)	0.67	0.23 / 1.11	31.06%	0.003	116	90	26
Maes et al. 2012 (IgA)	0.71	0.27 / 1.15	30.92%	0.002	116	90	26
Maes et al. 2008 (IgA)	0.95	0.38 / 1.52	18.45%	0.001	51	28	23
Unclear	0.73	0.48 / 0.97	100.00%	0.000	334	236	98

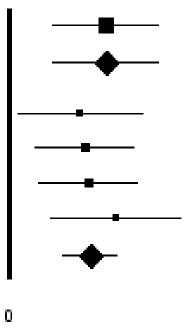

	Q	df	Sig.	I^2	T^2
Unclear	0.76	3	0.859	0.00	0.00

A-1-AT

	ES	95% CI	W	Sig.	N	N1	N2
Karaoulanis et al. 2014	0.38	-0.11 / 0.88	100.00%	0.129	65	39	26
Moderate to Severe	0.38	-0.11 / 0.88	100.00%	0.129	65	39	26
Bai et al. 2021	-0.10	-0.58 / 0.39	49.68%	0.699	106	20	86
Papakostas et al. 2013	0.87	0.41 / 1.32	50.32%	0.000	79	36	43
Severe	0.39	-0.55 / 1.33	100.00%	0.419	185	56	129

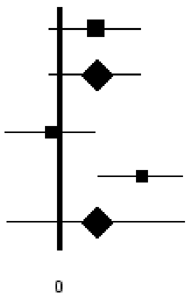

	Q	df	Sig.	I^2	T^2
Severe	8	1	0.005	87.50	0.40

Bibliography

1. A. Fasano: Zonulin and its regulation of intestinal barrier function: the biological door to inflammation, autoimmunity, and cancer. *Physiol Rev* 91(1), 151–175 (2011).
2. W.-T. Kuo, L. Shen, L. Zuo, N. Shashikanth, M.L.D.M. Ong, L. Wu, J. Zha, K.L. Edelblum, Y. Wang, Y. Wang, S.P. Nilsen, J.R. Turner: Inflammation-induced Occludin Downregulation Limits Epithelial Apoptosis by Suppressing Caspase-3 Expression. *Gastroenterology* 157(5), 1323–1337 (2019).
3. J.M. Wells, R.J. Brummer, M. Derrien, T.T. MacDonald, F. Troost, P.D. Cani, V. Theodorou, J. Dekker, A. Méheust, W.M. de Vos, A. Mercenier, A. Nauta, C.L. Garcia-Rodenas: Homeostasis of the gut barrier and potential biomarkers. *Am J Physiol Gastrointest Liver Physiol* 312(3), G171–G193 (2017).
4. M.R. Konikoff, L.A. Denson: Role of fecal calprotectin as a biomarker of intestinal inflammation in inflammatory bowel disease. *Inflamm Bowel Dis* 12(6), 524–534 (2006).
5. E.R. Cobo, K. Chadee: Antimicrobial Human β-Defensins in the Colon and Their Role in Infectious and Non-Infectious Diseases. *Pathogens* 2(1), 177–192 (2013).
6. K. Becker, T. Frieling, D. Häussinger: Quantification of fecal alpha 1-antitrypsin excretion for assessment of inflammatory bowel diseases. *Eur J Med Res* 3(1–2), 65–70 (1998).
7. B. Seethaler, M. Basrai, A.M. Neyrinck, J.-A. Nazare, J. Walter, N.M. Delzenne, S.C. Bischoff: Biomarkers for assessment of intestinal permeability in clinical practice. *Am J Physiol Gastrointest Liver Physiol* 321(1), G11–G17 (2021).
8. T.J. Faustmann, D. Kamp, S. Räuber, N. Melzer, L. Schilbach: sCD14, a marker of immune-inflammation can help to distinguish between psychotic disorders with and without disordered social interaction. *Medical Hypotheses* 181111190 (2023).
9. F. Vernia, A. Viscido, M. Di Ruscio, G. Stefanelli, M. Valvano, G. Latella: Fecal Lactoferrin and Other Putative Fecal Biomarkers in Crohn's Disease: Do They Still Have a Potential Clinical Role? *Digestion* 102(6), 833–844 (2021).
10. J.C. Hurley: Towards clinical applications of anti-endotoxin antibodies; a re-appraisal of the disconnect. *Toxins (Basel)* 5(12), 2589–2620 (2013).
11. X. Wang, P.J. Quinn: Endotoxins: Lipopolysaccharides of Gram-Negative Bacteria. In: Wang, X., Quinn, P.J. (eds.) Endotoxins: Structure, Function and Recognition. 3–25. *Springer Netherlands*, Dordrecht (2010).
12. B. Bertani, N. Ruiz: Function and Biogenesis of Lipopolysaccharides. *EcoSal Plus* 8(1), (2018).
13. L. Ohlsson, A. Gustafsson, E. Lavant, K. Suneson, L. Brundin, Å. Westrin, L. Ljunggren, D. Lindqvist: Leaky gut biomarkers in depression and suicidal behavior. *Acta Psychiatr Scand* 139(2), 185–193 (2019).
14. M.A. Alvarez-Mon, A.M. Gómez, A. Orozco, G. Lahera, M.D. Sosa, D. Diaz, E. Auba, A. Albillos, J. Monserrat, M. Alvarez-Mon: Abnormal Distribution and Function of Circulating Monocytes and Enhanced Bacterial Translocation in Major Depressive Disorder. *Frontiers in Psychiatry* 10 (2019).
15. M.A. Alvarez-Mon, A.M. Gomez-Lahoz, A. Orozco, G. Lahera, M.D. Sosa-Reina, D. Diaz, A. Albillos, J. Quintero, P. Molero, J. Monserrat, M. Alvarez-Mon: Blunted Expansion of Regulatory T Lymphocytes Is Associated With Increased Bacterial Translocation in Patients With Major Depressive Disorder. *Frontiers in Psychiatry* 11 (2021).
16. H. Wu, J. Wang, T. Teng, B. Yin, Y. He, Y. Jiang, X. Liu, Y. Yu, X. Li, X. Zhou: Biomarkers of intestinal permeability and blood-brain barrier permeability in adolescents with major depressive disorder. *Journal of Affective Disorders* 323659–666 (2023).
17. E. Osuna, J. Baumgartner, O. Wunderlin, S. Emery, M. Albermann, N. Baumgartner, K. Schmeck, S. Walitza, M. Strumberger, M. Hersberger, M.B. Zimmermann, I. Häberling, G. Berger, I. Herter-Aeberli: Iron status in Swiss adolescents with paediatric major depressive disorder and

healthy controls: a matched case-control study. *European journal of nutrition* 63(3), 951–963 (2024).
18. G.I. Papakostas, R.C. Shelton, G. Kinrys, M.E. Henry, B.R. Bakow, S.H. Lipkin, B. Pi, L. Thurmond, J.A. Bilello: Assessment of a multi-assay, serum-based biological diagnostic test for major depressive disorder: A Pilot and Replication Study. *Molecular Psychiatry* 18(3), 332–339 (2013).
19. J.Z. Brouillet, M. Boltri, A. Lengvenyte, M. Lajnef, J.-R. Richard, C. Barrau, R. Strumila, M. Coyac, C.-L. Wu, W. Boukouaci, S. Sugunasabesan, J. Bouassida, S. Guillaume, M. Sénèque, E. Olié, M. Leboyer, P. Courtet, R. Tamouza: Association of markers of inflammation and intestinal permeability in suicidal patients with major mood disorders. *Journal of Affective Disorders Reports* 14 (2023).
20. J.C. Stewart, B.M. Polanka, K.A. So-Armah, J.R. White, S.K. Gupta, S. Kundu, C.-C.H. Chang, M.S. Freiberg: Associations of Total, Cognitive/Affective, and Somatic Depressive Symptoms and Antidepressant Use With Cardiovascular Disease-Relevant Biomarkers in HIV: Veterans Aging Cohort Study. *Psychosomatic medicine* 82(5), 461–470 (2020).
21. D. Just, A.J. Rasmusson, P. Nilsson, M. Noreland, E. Malmström, P. Brodin, A. Månberg, J.L. Cunningham: Autoantibodies against the C-terminus of Lipopolysaccharide binding protein are elevated in young adults with psychiatric disease. *Psychoneuroendocrinology* 126105162–105162 (2021).
22. R. Musil, M.J. Schwarz, M. Riedel, S. Dehning, A. Cerovecki, I. Spellmann, V. Arolt, N. Müller: Elevated macrophage migration inhibitory factor and decreased transforming growth factor-beta levels in major depression--no influence of celecoxib treatment. *Journal of affective disorders* 134(1–3), 217–225 (2011).
23. S. Zengil, E. Laloğlu: Evaluation of Serum Zonulin and Occludin Levels in Bipolar Disorder. *Psychiatry Investigation* 20(4), 382–389 (2023).
24. S. Lee, M.V. Tejesvi, E. Hurskainen, O. Aasmets, J. Plaza-Díaz, S. Franks, L. Morin-Papunen, J.S. Tapanainen, T.S. Ruuska, S. Altmäe, E. Org, A. Salumets, R.K. Arffman, T.T. Piltonen: Gut bacteriome and mood disorders in women with PCOS. *Human reproduction (Oxford, England)* 39(6), 1291–1302 (2024).
25. M. Maes, M. Kubera, J.-C. Leunis, M. Berk, M. Geffard, E. Bosmans: In depression, bacterial translocation may drive inflammatory responses, oxidative and nitrosative stress (O&NS), and autoimmune responses directed against O&NS-damaged neoepitopes. *Acta psychiatrica Scandinavica* 127(5), 344–354 (2013).
26. S. Bai, L. Fang, J. Xie, H. Bai, W. Wang, J.-J. Chen: Potential biomarkers for diagnosing major depressive disorder patients with suicidal ideation. *Journal of Inflammation Research* 14495–503 (2021).
27. S.E. Karaoulanis, K.A. Rizouli, A.A. Rizoulis, N.V. Angelopoulos: Lack of association of acute phase response proteins with hormone levels and antidepressant medication in perimenopausal depression. *BMC Psychiatry* 14(1), (2014).
28. A. Maget, N. Dalkner, C. Hamm, S.A. Bengesser, F.T. Fellendorf, M. Platzer, R. Queissner, A. Birner, M. Lenger, S. Mörkl, A. Kohlhammer-Dohr, A. Rieger, M. Seidl, L. Mendel, T. Färber, L. Wetzlmair, K. Schwalsberger, D.V. Amberger-Otti, H. Schöggl, T. Lahousen, B. Leitner-Afschar, R. Unterweger, S. Zelzer, H. Mangge, E.Z. Reininghaus: Sex differences in zonulin in affective disorders and associations with current mood symptoms. *Journal of affective disorders* 294441–446 (2021).
29. M. Maes, M. Kubera, J.-C. Leunis: The gut-brain barrier in major depression: intestinal mucosal dysfunction with an increased translocation of LPS from gram negative enterobacteria (leaky gut) plays a role in the inflammatory pathophysiology of depression. *Neuro endocrinology letters* 29(1), 117–124 (2008).
30. M. Maes, D. Simeonova, D. Stoyanov, J.-C. Leunis: Upregulation of the nitrosylome in bipolar disorder type 1 (BP1) and major depression, but not BP2: Increased IgM antibodies to

nitrosylated conjugates are associated with indicants of leaky gut. *Nitric oxide : biology and chemistry* 9167–76 (2019).
31. J. Zhong, J. Chen, M. Cao, L. Fang, Z. Wang, J. Liao, D. Chen, X. Zhang, J. Guo, L. Zhao, C. Zhou: Elevated plasma intestinal fatty acid binding protein and aberrant lipid metabolism predict post-stroke depression. *Heliyon* 8(11), e11848 (2022).
32. V.C.-H. Chen, S.-I. Wu: An exploratory analysis on the association between suicidal ideation and the microbiome in patients with or without major depressive disorder, https://www.researchsquare.com/article/rs-4119552/v1,%20(2024)., (2024).
33. L. Wang, C. Chunyou, J. Zhu, X. Bao, X. Tao: Prediction of post-stroke depression with combined blood biomarkers IL-6, TNF-a, and fatty acid binding protein: A prospective study. *J Med Biochem* 42(4), 638–644 (2023).
