# Supplementary material for: Leaky Gut Biomarkers as Predictors of Depression and Suicidal Risk: A Systematic Review and Meta-Analysis
Source: Diagnostics (Basel). 2025 Jul 1;15(13):1683. doi: 10.3390/diagnostics15131683 (PMC12249198; doi:10.3390/diagnostics15131683)
Supplement: Supplementary file 1 [file diagnostics-15-01683-s001.zip › Supplement S3 LGM 12.06.pdf]

Leaky Gut Biomarkers as Predictors of Depression and Suicidal Risk: A Systematic Review and Meta-Analysis – Supplement S3

Included and Excluded Studies, with reasons

Studies identified from the primary search

Title	Reason (Explanation)
1. Maes M. A new case definition of Neuro-Inflammatory and Oxidative Fatigue (NIOF), a neuroprogressive disorder, formerly known as chronic fatigue syndrome or Myalgic Encephalomyelitis: results of multivariate pattern recognition methods and external validation by neuro-immune biomarkers. Neuro Endocrinol Lett. 2015;36(4):320-9. PMID: 26454487.	Excluded- Lack of data
2. Polster A, Öhman L, Tap J, Derrien M, Le Nevé B, Sundin J, Törnblom H, Cvijovic M, Simrén M. A novel stepwise integrative analysis pipeline reveals distinct microbiota-host interactions and link to symptoms in irritable bowel syndrome. Sci Rep. 2021 Mar 9;11(1):5521. doi: 10.1038/s41598-021-84686-9. PMID: 33750831; PMCID: PMC7943560.	Excluded- Lack of data<br>(Specific data for depressive patients)
3. Farhangi MA, Javid AZ, Sarmadi B, Karimi P, Dehghan P. A randomized controlled trial on the efficacy of resistant dextrin, as functional food, in women with type 2 diabetes: Targeting the hypothalamic-pituitary-adrenal axis and immune system. Clin Nutr. 2018 Aug;37(4):1216-1223. doi: 10.1016/j.clnu.2017.06.005. Epub 2017 Jun 10. PMID: 28669666.	Excluded- Lack of data<br>(Correlation between DASS Score and LPS levels)
4. Janssen LM, Rezazadeh Ardabili A, Romberg-Camps MJL, Winkens B, van den Broek RJ, Hulst J, Verwijs HJA, Keszthelyi D, Jonkers DMAE, van Bodegraven AA, Pierik MJ, Mujagic Z. Abdominal pain in patients with inflammatory bowel disease in remission: A prospective study on contributing factors. Aliment Pharmacol Ther. 2023 Nov;58(10):1041-1051. doi: 10.1111/apt.17718. Epub 2023 Sep 19. PMID: 37724651.	Excluded- Lack of data<br>(Correlation between HADS-D and faecal calprotectin levels)

5. Alvarez-Mon MA, Gómez AM, Orozco A, Lahera G, Sosa MD, Diaz D, Auba E, Albillos A, Monserrat J, Alvarez-Mon M. Abnormal Distribution and Function of Circulating Monocytes and Enhanced Bacterial Translocation in Major Depressive Disorder. Front Psychiatry. 2019 Nov 15;10:812. doi: 10.3389/fpsyt.2019.00812. PMID: 31803077; PMCID: PMC6873610.	Included in systematic review and meta-analysis (Additional data requested. After two attempts, the data were manually extracted using web-based tools)
6. Maes M, Rachayon M, Jirakran K, Sodsai P, Klinchanhom S, Debnath M, Basta-Kaim A, Kubera M, Almulla AF, Sughondhabirom A. Adverse Childhood Experiences Predict the Phenome of Affective Disorders and These Effects Are Mediated by Staging, Neuroimmunotoxic and Growth Factor Profiles. Cells. 2022 May 7;11(9):1564. doi: 10.3390/cells11091564. PMID: 35563878; PMCID: PMC9105661.	Excluded– Wrong focus<br>(Focus different from the one of interest)
7. Chojnacki C, Konrad P, Błońska A, Medrek-Socha M, Przybylowska-Sygut K, Chojnacki J, Poplawski T. Altered Tryptophan Metabolism on the Kynurenine Pathway in Depressive Patients with Small Intestinal Bacterial Overgrowth. Nutrients. 2022 Aug 6;14(15):3217. doi: 10.3390/nu14153217. PMID: 35956393; PMCID: PMC9370164.	Included in systematic review
8. Wardle RA, Thapaliya G, Nowak A, Radford S, Dalton M, Finlayson G, Moran GW. An Examination of Appetite and Disordered Eating in Active Crohn's Disease. J Crohns Colitis. 2018 Jun 28;12(7):819-825. doi: 10.1093/ecco-jcc/jjy041. PMID: 29617753.	Excluded- Lack of data<br>(Correlation between Hospital Depression Scale score and faecal calprotectin levels)
9. Wardle RA, Thapaliya G, Nowak A, Radford S, Dalton M, Finlayson G, Moran GW. An Examination of Appetite and Disordered Eating in Active Crohn's Disease. J Crohns Colitis. 2018 Jun 28;12(7):819-825. doi: 10.1093/ecco-jcc/jjy041. PMID: 29617753.	Excluded- Lack of data<br>(Correlation between Hospital Depression Scale score and faecal calprotectin (µg/g) levels for IBS and IBD patients)
10. Liśkiewicz P, Kaczmarczyk M, Misiak B, Wroński M, Bąba-Kubiś A, Skonieczna-Żydecka K, Marlicz W, Bieńkowski P, Misera A, Pełka-Wysiecka J, Kucharska-Mazur J, Konopka A, Łoniewski I, Samochowiec	Included in systematic review

J. Analysis of gut microbiota and intestinal integrity markers of inpatients with major depressive disorder. Prog Neuropsychopharmacol Biol Psychiatry. 2021 Mar 2;106:110076. doi: 10.1016/j.pnpbp.2020.110076. Epub 2020 Aug 19. PMID: 32827611.	
11. Liśkiewicz P, Kaczmarczyk M, Misiak B, Wroński M, Bąba-Kubiś A, Skonieczna-Żydecka K, Marlicz W, Bieńkowski P, Misera A, Pełka-Wysiecka J, Kucharska-Mazur J, Konopka A, Łoniewski I, Samochowiec J. Analysis of gut microbiota and intestinal integrity markers of inpatients with major depressive disorder. Prog Neuropsychopharmacol Biol Psychiatry. 2021 Mar 2;106:110076. doi: 10.1016/j.pnpbp.2020.110076. Epub 2020 Aug 19. PMID: 32827611.	Excluded- Lack of data<br>(Correlation between Hamilton Depression Scale score and biomarkers such as zonulin, calprotectin, , IFABP and LPS at baseline)
12. Kudinova AY, Gano A, James KM, Lawlor C, Deak T, Gibb BE. Anhedonia and Increased Evoked Immune Response. Brain Behav Immun Health. 2020 Jul;6:100090. doi: 10.1016/j.bbih.2020.100090. Epub 2020 Jun 5. PMID: 33718895; PMCID: PMC7954217.	Excluded– Wrong focus<br>(Focus different from the one of interest)
13. Freed RD, Mehra LM, Laor D, Patel M, Alonso CM, Kim-Schulze S, Gabbay V. Anhedonia as a clinical correlate of inflammation in adolescents across psychiatric conditions. World J Biol Psychiatry. 2019 Nov;20(9):712-722. doi: 10.1080/15622975.2018.1482000. Epub 2018 Aug 16. PMID: 29843560; PMCID: PMC6377856.	Excluded– Wrong focus<br>(Focus different from the one of interest)
14. Kubera M, Lin AH, Kenis G, Bosmans E, van Bockstaele D, Maes M. Anti-Inflammatory effects of antidepressants through suppression of the interferon-gamma/interleukin-10 production ratio. J Clin Psychopharmacol. 2001 Apr;21(2):199-206. doi: 10.1097/00004714-200104000-00012. PMID: 11270917.	Excluded– Wrong focus<br>(Focus different from the one of interest)
15. Kranaster L, Hoyer C, Aksay SS, Bumb JM, Müller N, Zill P, Schwarz MJ, Sartorius A. Antidepressant efficacy of electroconvulsive therapy is associated with a reduction of the innate cellular	Excluded- Lack of data<br>(Correlation between Hamilton Depression Scale 21 score and sCD14 at baseline)

immune activity in the cerebrospinal fluid in patients with depression. World J Biol Psychiatry. 2018 Aug;19(5):379-389. doi: 10.1080/15622975.2017.1355473. Epub 2017 Aug 9. PMID: 28714751.	
16. Miguel-Hidalgo JJ, Whittom A, Villarreal A, Soni M, Meshram A, Pickett JC, Rajkowska G, Stockmeier CA. Apoptosis-related proteins and proliferation markers in the orbitofrontal cortex in major depressive disorder. J Affect Disord. 2014 Apr;158:62-70. doi: 10.1016/j.jad.2014.02.010. Epub 2014 Feb 10. PMID: 24655767; PMCID: PMC3996705.	Excluded– Wrong focus<br>(Focus different from the one of interest)
17. Papakostas GI, Shelton RC, Kinrys G, Henry ME, Bakow BR, Lipkin SH, Pi B, Thurmond L, Bilello JA. Assessment of a multi-assay, serum-based biological diagnostic test for major depressive disorder: a pilot and replication study. Mol Psychiatry. 2013 Mar;18(3):332-9. doi: 10.1038/mp.2011.166. Epub 2011 Dec 13. PMID: 22158016.	Included in systematic review and meta-analysis
18. Komorniak N, Martynova-Van Kley A, Nalian A, Wroński M, Kaseja K, Kowalewski B, Kaźmierczak-Siedlecka K, Łoniewski I, Kaczmarczyk M, Podsiadło K, Bogdański P, Palma J, Stachowska E. Association between Fecal Microbiota, SCFA, Gut Integrity Markers and Depressive Symptoms in Patients Treated in the Past with Bariatric Surgery-The Cross-Sectional Study. Nutrients. 2022 Dec 17;14(24):5372. doi: 10.3390/nu14245372. PMID: 36558532; PMCID: PMC9781380.	Excluded- Lack of data<br>(Correlation between Beck's scale and Hamilton's scale score and levels of LPS, LBP, occludin, zonulin)
19. Rajkovaca Latic I, Popovic Z, Mijatovic K, Sahinovic I, Pekic V, Vucic D, Cosic V, Miskic B, Tomic S. Association of intestinal inflammation and permeability markers with clinical manifestations of Parkinson's disease. Parkinsonism Relat Disord. 2024 Jun;123:106948. doi: 10.1016/j.parkreldis.2024.106948. Epub 2024 Mar 27. PMID: 38554664.	Included in systematic review
20. Brouillet, J. Z., Boltri, M., Lengvenyte, A., Lajnef, M., Richard, J. R., Barrau, C., ... & Tamouza, R. (2023). Association of	Included in systematic review and meta-analysis

markers of inflammation and intestinal permeability in suicidal patients with major mood disorders. Journal of Affective Disorders Reports, 14, 100624.	
21. Đogaš T, Novak I, Babić M, Vučković M, Tandara L, Radić J. Associations of Serum Calprotectin, Arterial Stiffness and Long COVID Symptoms in Dalmatian Kidney Transplant Recipients. Viruses. 2023 Aug 21;15(8):1776. doi: 10.3390/v15081776. PMID: 37632118; PMCID: PMC10458603.	Excluded- Lack of data<br>(Correlation between depression at EQ-5D-5L and levels of serum calprotectin)
22. Stewart JC, Polanka BM, So-Armah KA, White JR, Gupta SK, Kundu S, Chang CH, Freiberg MS. Associations of Total, Cognitive/Affective, and Somatic Depressive Symptoms and Antidepressant Use With Cardiovascular Disease-Relevant Biomarkers in HIV: Veterans Aging Cohort Study. Psychosom Med. 2020 Jun;82(5):461-470. doi: 10.1097/PSY.0000000000000808. PMID: 32282648; PMCID: PMC7282983.	Included in systematic review and meta-analysis
23. Bharti AR, McCutchan JA, Umlauf A, Okwuegbuna OK, Letendre S, Cherner M, Burdo T, Jumare J, Williams K, Blattner W, Royal W. Asymptomatic Malaria Co-infection of HIV-Infected Adults in Nigeria: Prevalence of and Impact on Cognition, Mood, and Biomarkers of Systemic Inflammation. J Acquir Immune Defic Syndr. 2021 Jan 1;86(1):91-97. doi: 10.1097/QAI.0000000000002516. PMID: 33021552; PMCID: PMC10742372.	Excluded- Lack of data<br>(Correlation between depression at Beck Depression Inventory and levels of serum sCD14)
24. Just D, Rasmusson AJ, Nilsson P, Noreland M, Malmström E, Brodin P, Månberg A, Cunningham JL. Autoantibodies against the C-terminus of Lipopolysaccharide binding protein are elevated in young adults with psychiatric disease. Psychoneuroendocrinology. 2021 Apr;126:105162. doi: 10.1016/j.psyneuen.2021.105162. Epub 2021 Feb 4. PMID: 33578084.	Included in systematic review and meta-analysis

25. Gracie DJ, Guthrie EA, Hamlin PJ, Ford AC. Bi-directionality of Brain-Gut Interactions in Patients With Inflammatory Bowel Disease. Gastroenterology. 2018 May;154(6):1635-1646.e3. doi: 10.1053/j.gastro.2018.01.027. Epub 2018 Jan 31. PMID: 29366841.	Excluded- Lack of data<br>(Correlation between depression at HADS and levels of fecal calprotectin)
26. Kranaster L, Hoyer C, Aksay SS, Bumb JM, Müller N, Zill P, Schwarz MJ, Moll N, Lutz B, Bindila L, Zerr I, Schmitz M, Blennow K, Zetterberg H, Haffner D, Leifheit-Nestler M, Ozbalci C, Janke C, Thiel M, Sartorius A. Biomarkers for Antidepressant Efficacy of Electroconvulsive Therapy: An Exploratory Cerebrospinal Fluid Study. Neuropsychobiology. 2019;77(1):13-22. doi: 10.1159/000491401. Epub 2018 Aug 17. PMID: 30121652.	Excluded- Lack of data<br>(Correlation between depression at HADS and levels of sCD14)
27. Wu H, Wang J, Teng T, Yin B, He Y, Jiang Y, Liu X, Yu Y, Li X, Zhou X. Biomarkers of intestinal permeability and blood-brain barrier permeability in adolescents with major depressive disorder. J Affect Disord. 2023 Feb 15;323:659-666. doi: 10.1016/j.jad.2022.11.058. Epub 2022 Dec 7. PMID: 36493942.	Included in systematic review and meta-analysis
28. Chahine A, Koru-Sengul T, Feaster DJ, Dilworth SE, Antoni MH, Klatt N, Roach ME, Pallikkuth S, Sharkey M, Salinas J, Stevenson M, Pahwa S, Fuchs D, Carrico AW. Blue Monday: Co-occurring Stimulant Use and HIV Persistence Predict Dysregulated Catecholamine Synthesis. J Acquir Immune Defic Syndr. 2021 Mar 1;86(3):353-360. doi: 10.1097/QAI.0000000000002560. PMID: 33165125; PMCID: PMC11215553.	Excluded- Lack of data<br>(Correlation between depression at 20-item Center for Epidemiologic Studies Depression and levels of sCD14 and of LBP at baseline)
29. Alvarez-Mon MA, Gomez-Lahoz AM, Orozco A, Lahera G, Sosa-Reina MD, Diaz D, Albillos A, Quintero J, Molero P, Monserrat J, Alvarez-Mon M. Blunted Expansion of Regulatory T Lymphocytes Is Associated With Increased Bacterial Translocation in Patients With Major Depressive Disorder. Front Psychiatry. 2021 Jan	Included in systematic review and meta-analysis<br>(Additional data requested. After two attempts, the data were manually extracted using web-based tools)

8;11:591962. doi: 10.3389/fpsyt.2020.591962. PMID: 33488424; PMCID: PMC7820111.	
30. Iordache MM, Tocia C, Aschie M, Dumitru A, Manea M, Cozaru GC, Petcu L, Vlad SE, Dumitru E, Chisoi A. Intestinal Permeability and Depression in Patients with Inflammatory Bowel Disease. J Clin Med. 2022 Aug 30;11(17):5121. doi: 10.3390/jcm11175121. PMID: 36079050; PMCID: PMC9457405..	Included systematic review
31. Hussain MA, Watson CW, Morgan EE, Heaton RK, Letendre SL, Jeste DV, Moore DJ, Iudicello JE. Combined effects of loneliness and inflammation on depression in people with HIV. J Neurovirol. 2023 Oct;29(5):538-554. doi: 10.1007/s13365-023-01145-z. Epub 2023 Aug 31. PMID: 37651083; PMCID: PMC10645641.	Included in systematic review
32. Simeonova D, Stoyanov D, Leunis JC, Murdjeva M, Maes M. Construction of a nitro-oxidative stress-driven, mechanistic model of mood disorders: A nomothetic network approach. Nitric Oxide. 2021 Jan 1;106:45-54. doi: 10.1016/j.niox.2020.11.001. Epub 2020 Nov 10. PMID: 33186727.	Excluded- Lack of data<br>(Levels of IgM and IgA directed to LPS in the 35 healthy controls, 47 major depressed, 29 Bipolar 1, and 25 Bipolar 2 patients.
33. Contreras-Rodriguez O, Reales-Moreno M, Fernández-Barrès S, Cimpean A, Arnoriaga-Rodríguez M, Puig J, Biarnés C, Motger-Albertí A, Cano M, Fernández-Real JM. Consumption of ultra-processed foods is associated with depression, mesocorticolimbic volume, and inflammation. J Affect Disord. 2023 Aug 15;335:340-348. doi: 10.1016/j.jad.2023.05.009. Epub 2023 May 18. PMID: 37207947.	Excluded- Lack of data<br>(Correlation between LBP levels and PHQ-9)
34. Grimstad T, Norheim KB, Kvaløy JT, Isaksen K, Leitao K, Carlsen A, Karlsen LN, Aabakken L, Omdal R. Conventional treatment regimens for ulcerative colitis alleviate fatigue - an observational cohort study. Scand J Gastroenterol. 2016 Oct;51(10):1213-9. doi: 10.1080/00365521.2016.1195869. Epub 2016 Jun 16. PMID: 27310658.	Excluded- Lack of data<br>(Correlation between Fecal Calprotectin levels and HADS-D score)
35. Asscher VER, Waars SN, van der Meulen-de Jong AE, Stuyt RJL, Baven-	Excluded- Lack of data

Pronk AMC, van der Marel S, Jacobs RJ, Haans JJL, Meijer LJ, Klijnsma-Slagboom JD, Duin MH, Peters MER, Lee-Kong FVYL, Provoost NE, Tijdeman F, van Dijk KT, Wieland MWM, Verstegen MGM, van der Meijs ME, Maan ADI, van Deudekom FJ, Mooijaart SP, Maljaars PWJ. Deficits in Geriatric Assessment Associate With Disease Activity and Burden in Older Patients With Inflammatory Bowel Disease. Clin Gastroenterol Hepatol. 2022 May;20(5):e1006-e1021. doi: 10.1016/j.cgh.2021.06.015. Epub 2021 Jun 19. Erratum in: Clin Gastroenterol Hepatol. 2023 Dec;21(13):3466. doi: 10.1016/j.cgh.2023.08.018. PMID: 34153476.	(Correlation between Fecal Calprotectin levels and Geriatric Depression Scale score)
36. Bandinelli F, Benucci M, Salaffi F, Manetti M, Infantino M, Damiani A, Manfredi M, Grossi V, Matucci A, Li Gobbi F, Marin G. Do new and old biomarkers of early undifferentiated arthritis correlate with Arthritis Impact Measurement Scales? Clin Exp Rheumatol. 2021 Jan-Feb;39(1):79-83. doi: 10.55563/clinexprheumatol/nqqx5k. Epub 2020 Mar 28. PMID: 32242813.	Excluded- Lack of data<br>(Correlation between Serum Calprotectinlevels and Depression score)
37. van den Brink G, Stapersma L, Bom AS, Rizopolous D, van der Woude CJ, Stuyt RJL, Hendriks DM, van der Burg JAT, Beukers R, Korpershoek TA, Theuns-Valks SDM, Utens EMWJ, Escher JC. Effect of Cognitive Behavioral Therapy on Clinical Disease Course in Adolescents and Young Adults With Inflammatory Bowel Disease and Subclinical Anxiety and/or Depression: Results of a Randomized Trial. Inflamm Bowel Dis. 2019 Nov 14;25(12):1945-1956. doi: 10.1093/ibd/izz073. PMID: 31050763; PMCID: PMC7006993.	Excluded- Lack of data<br>(Correlation between Fecal Calprotectin levels and CDI/ BDI-II)
38. Negm M, Bahaa A, Farrag A, Lithy RM, Badary HA, Essam M, Kamel S, Sakr M, Abd El Aaty W, Shamkh M, Basiony A, Dawoud I, Shehab H. Effect of Ramadan intermittent fasting on inflammatory markers, disease severity, depression, and quality of life in patients with inflammatory bowel diseases: A	Excluded- Lack of data<br>(Correlation between Fecal Calprotectin levels and Hamilton depression scale questionnaire score)

prospective cohort study. BMC Gastroenterol. 2022 Apr 24;22(1):203. doi: 10.1186/s12876-022-02272-3. PMID: 35462542; PMCID: PMC9036734.	
39. Choi J, Kim JH, Park M, Lee HJ. Effects of Flavonoid-Rich Orange Juice Intervention on Major Depressive Disorder in Young Adults: A Randomized Controlled Trial. Nutrients. 2022 Dec 28;15(1):145. doi: 10.3390/nu15010145. PMID: 36615801; PMCID: PMC9823945.	Excluded- Lack of data<br>(Correlation between Fatty Acid Binding Protein 2. Zonuline, lipopolysaccharide, and claudin-5 levels and BDI-II, CES-D. HAMD-17 at baseline)
40. Vaghef-Mehrabani E, Harouni R, Behrooz M, Ranjbar F, Asghari-Jafarabadi M, Ebrahimi-Mameghani M. Effects of inulin supplementation on inflammatory biomarkers and clinical symptoms of women with obesity and depression on a calorie-restricted diet: a randomised controlled clinical trial. Br J Nutr. 2023 Jun 14;129(11):1897-1907. doi: 10.1017/S000711452200232X. Epub 2022 Sep 5. PMID: 36059088.	Excluded- Lack of data<br>(Correlation between lipopolysaccharide, Zonulin levels and HDRS-17 score at baseline for both groups)
41. Musil R, Schwarz MJ, Riedel M, Dehning S, Cerovecki A, Spellmann I, Arolt V, Müller N. Elevated macrophage migration inhibitory factor and decreased transforming growth factor-beta levels in major depression--no influence of celecoxib treatment. J Affect Disord. 2011 Nov;134(1-3):217-25. doi: 10.1016/j.jad.2011.05.047. Epub 2011 Jun 17. PMID: 21684012.	Included in systematic review and meta-analysis
42. Oktayoglu P, Mete N, Caglayan M, Bozkurt M, Bozan T, Em S, Nas K. Elevated serum levels of calprotectin (MRP8/MRP14) in patients with Behçet's disease and its association with disease activity and quality of life. Scand J Clin Lab Invest. 2015 Apr;75(2):106-12. doi: 10.3109/00365513.2014.984319. Epub 2014 Dec 4. PMID: 25471894.	Included in systematic review
43. Madison AA, Andridge R, Padin AC, Wilson S, Bailey MT, Alfano CM, Povoski SP, Lipari AM, Agnese DM, Carson WE, Malarkey WB, Kiecolt-Glaser JK. Endotoxemia coupled with heightened inflammation predicts future depressive symptoms.	Included in systematic review

Psychoneuroendocrinology. 2020 Dec;122:104864. doi: 10.1016/j.psyneuen.2020.104864. Epub 2020 Oct 8. PMID: 33166799; PMCID: PMC7721058.	
44. Bellei E, Bergamini S, Monari E, Tomasi A, Koseoglu M, Topaloglu Tuac S, Ozben S. Evaluation of potential cardiovascular risk protein biomarkers in high severity restless legs syndrome. J Neural Transm (Vienna). 2019 Oct;126(10):1313-1320. doi: 10.1007/s00702-019-02051-7. Epub 2019 Jul 23. PMID: 31338581.	Included in systematic review
45. Zengil S, Laloğlu E. Evaluation of Serum Zonulin and Occludin Levels in Bipolar Disorder. Psychiatry Investig. 2023 Apr;20(4):382-389. doi: 10.30773/pi.2022.0234. Epub 2023 Apr 20. PMID: 37098666; PMCID: PMC10151659.	Included in systematic review and meta-analysis<br>(Additional data requested but not received:<br>For depressive patients: Age, %F, HDRS score, BMI, Smoking, Alcohol<br>For suicide attempters: Age, %F, HDRS score, BMI, Smoking, Alcohol<br>Setting, Period)
46. Aydın O, Kocabaş T, Sarandöl A, Taştan İ, Onur E, Aydemir Ö, Esen-Danacı A. Examination of plasma zonulin levels in bipolar I disorder: a case-control study with follow-up. J Neural Transm (Vienna). 2020 Oct;127(10):1419-1426. doi: 10.1007/s00702-020-02234-7. Epub 2020 Jul 21. PMID: 32696242.	Excluded- Lack of data<br>(All information that was provided for the entire group of Bipolar I patients should have been split and provided for depressive patients)
47. Maes M, Vasupanrajit A, Jirakran K, Klomkliew P, Chanchaem P, Tunvirachaisakul C, Payungporn S. Exploration of the Gut Microbiome in Thai Patients with Major Depressive Disorder Shows a Specific Bacterial Profile with Depletion of the Ruminococcus Genus as a Putative Biomarker. Cells. 2023 Apr 25;12(9):1240. doi: 10.3390/cells12091240. PMID: 37174640; PMCID: PMC10177051.	Excluded– Wrong focus<br>(Focus different from the one of interest)
48. Hasebe K, Mohebbi M, Gray L, Walker AJ, Bortolasci CC, Turner A, Berk M, Walder K, Maes M, Kanchanatawan B, Ashton MM, Berk L, Ng CH, Malhi GS, Singh AB, Dean OM. Exploring interleukin-6, lipopolysaccharide-binding protein and brain-derived neurotrophic factor following 12 weeks of adjunctive minocycline treatment for depression. Acta Neuropsychiatr. 2022	Excluded- Lack of data<br>(Correlation between Serum LBP concentrations and parameters)

Aug;34(4):220-227. doi: 10.1017/neu.2021.44. Epub 2021 Dec 23. PMID: 34937590.	
49. Grimstad T, Norheim KB, Isaksen K, Leitao K, Hetta AK, Carlsen A, Karlsen LN, Skoie IM, Gøransson L, Harboe E, Aabakken L, Omdal R. Fatigue in Newly Diagnosed Inflammatory Bowel Disease. J Crohns Colitis. 2015 Sep;9(9):725-30. doi: 10.1093/ecco-jcc/jjv091. Epub 2015 May 19. PMID: 25994356.	Excluded- Lack of data<br>(Correlation between faecal calprotectin concentrations and HADS-D)
50. Uhlir V, Stallmach A, Grunert PC. Fatigue in patients with inflammatory bowel disease-strongly influenced by depression and not identifiable through laboratory testing: a cross-sectional survey study. BMC Gastroenterol. 2023 Aug 22;23(1):288. doi: 10.1186/s12876-023-02906-0. PMID: 37608313; PMCID: PMC10463723.	Excluded- Lack of data<br>(Correlation between faecal calprotectin concentrations and HADS-D)
51. Mikocka-Walus A, Hughes PA, Bampton P, Gordon A, Campaniello MA, Mavrangelos C, Stewart BJ, Esterman A, Andrews JM. Fluoxetine for Maintenance of Remission and to Improve Quality of Life in Patients with Crohn's Disease: a Pilot Randomized Placebo-Controlled Trial. J Crohns Colitis. 2017 Apr 1;11(4):509-514. doi: 10.1093/ecco-jcc/jjw165. PMID: 27664274; PMCID: PMC5881791.	Excluded- Lack of data<br>(Correlation between faecal calprotectin concentrations and HADS-D)
52. Oliveira R, Martins V, de Sousa HT, Roseira J. Food-Related Quality of Life and Its Predictors in Inflammatory Bowel Disease. Dig Dis Sci. 2024 May;69(5):1793-1802. doi: 10.1007/s10620-024-08333-9. Epub 2024 Mar 8. PMID: 38457117.	Excluded - Wrong design<br>(Design study not adequate for our meta-analysis)
53. Lee S, Tejesvi MV, Hurskainen E, Aasmets O, Plaza-Díaz J, Franks S, Morin-Papunen L, Tapanainen JS, Ruuska TS, Altmäe S, Org E, Salumets A, Arffman RK, Piltonen TT. Gut bacteriome and mood disorders in women with PCOS. Hum Reprod. 2024 Jun 3;39(6):1291-1302. doi: 10.1093/humrep/deae073. PMID: 38614956; PMCID: PMC11145006.	Included in systematic review and meta-analysis<br>(Additional data requested but not received: for no-MD and MD: Depression score at BDI-II)

54. Bai S, Bai H, Li D, Zhong Q, Xie J, Chen JJ. Gut Microbiota-Related Inflammation Factors as a Potential Biomarker for Diagnosing Major Depressive Disorder. Front Cell Infect Microbiol. 2022 Mar 15;12:831186. doi: 10.3389/fcimb.2022.831186. PMID: 35372107; PMCID: PMC8965553.	Included in systematic review (Additional data requested but not received: for HCs MDD patients: levels of AAT)
55. Caso JR, MacDowell KS, González-Pinto A, García S, de Diego-Adeliño J, Carceller-Sindreu M, Sarramea F, Caballero-Villarraso J, Gracia-García P, De la Cámara C, Agüera L, Gómez-Lus ML, Alba C, Rodríguez JM, Leza JC. Gut microbiota, innate immune pathways, and inflammatory control mechanisms in patients with major depressive disorder. Transl Psychiatry. 2021 Dec 21;11(1):645. doi: 10.1038/s41398-021-01755-3. PMID: 34934041; PMCID: PMC8692500.	Excluded- Lack of data
56. Kim-Chang JJ, Donovan K, Loop MS, Hong S, Fischer B, Venturi G, Garvie PA, Kohn J, Rendina HJ, Woods SP, Goodenow MM, Nichols SL, Sleasman JW; Adolescent Medicine Trials Network for HIV/AIDS Interventions. Higher soluble CD14 levels are associated with lower visuospatial memory performance in youth with HIV. AIDS. 2019 Dec 1;33(15):2363-2374. doi: 10.1097/QAD.0000000000002371. Erratum in: AIDS. 2020 Feb 1;34(2):333. doi: 10.1097/01.aids.0000617292.75887.1a. PMID: 31764101; PMCID: PMC6905124.	Excluded- Lack of data<br>(Correlation depressive scores and sCD14)
57. Anderson AM, Bhondoekhan F, Curanovic D, Connelly MA, Otvos JD, Post WS, Michos ED, Stosor V, Levine A, Seaberg E, Weinstein AM, Becker JT. Higher Soluble CD163 in Blood Is Associated With Significant Depression Symptoms in Men With HIV. J Acquir Immune Defic Syndr. 2022 Nov 1;91(3):325-333. doi: 10.1097/QAI.0000000000003063. PMID: 35969468; PMCID: PMC9588493.	Excluded- Other reasons<br>(Paper not available for our Institution)
58. Jonefjäll B, Öhman L, Simrén M, Strid H. IBS-like Symptoms in Patients with	Excluded- Lack of data<br>(Correlation depressive scores and calprotectin)

Ulcerative Colitis in Deep Remission Are Associated with Increased Levels of Serum Cytokines and Poor Psychological Well-being. Inflamm Bowel Dis. 2016 Nov;22(11):2630-2640. doi: 10.1097/MIB.0000000000000921. PMID: 27636379.	
59. Schwarz E, Guest PC, Rahmoune H, Harris LW, Wang L, Leweke FM, Rothermundt M, Bogerts B, Koethe D, Kranaster L, Ohrmann P, Suslow T, McAllister G, Spain M, Barnes A, van Beveren NJ, Baron-Cohen S, Steiner J, Torrey FE, Yolken RH, Bahn S. Identification of a biological signature for schizophrenia in serum. Mol Psychiatry. 2012 May;17(5):494-502. doi: 10.1038/mp.2011.42. Epub 2011 Apr 12. PMID: 21483431.	Excluded- Lack of data (AAT for MDD patients)
60. Keane JM, Khashan AS, McCarthy FP, Kenny LC, Collins JM, O'Donovan S, Brown J, Cryan JF, Dinan TG, Clarke G, O'Mahony SM. Identifying a biological signature of prenatal maternal stress. JCI Insight. 2021 Jan 25;6(2):e143007. doi: 10.1172/jci.insight.143007. PMID: 33301421; PMCID: PMC7934857.	Excluded- Lack of data
61. Roomruangwong C, Kanchanatawan B, Sirivichayakul S, Anderson G, Carvalho AF, Duleu S, Geffard M, Maes M. IgM-mediated autoimmune responses to oxidative specific epitopes, but not nitrosylated adducts, are significantly decreased in pregnancy: association with bacterial translocation, perinatal and lifetime major depression and the tryptophan catabolite (TRYCAT) pathway. Metab Brain Dis. 2017 Oct;32(5):1571-1583. doi: 10.1007/s11011-017-0040-2. Epub 2017 Jun 9. PMID: 28600633.	Excluded- Lack of data
62. Maes M, Kubera M, Leunis JC, Berk M, Geffard M, Bosmans E. In depression, bacterial translocation may drive inflammatory responses, oxidative and nitrosative stress (O&NS), and autoimmune responses directed against O&NS-damaged neoepitopes. Acta Psychiatr Scand. 2013 May;127(5):344-54. doi: 10.1111/j.1600-	Included in systematic review and meta-analysis

0447.2012.01908.x. Epub 2012 Aug 17. PMID: 22900942.	
63. Stevens BR, Goel R, Seungbum K, Richards EM, Holbert RC, Pepine CJ, Raizada MK. Increased human intestinal barrier permeability plasma biomarkers zonulin and FABP2 correlated with plasma LPS and altered gut microbiome in anxiety or depression. Gut. 2018 Aug;67(8):1555-1557. doi: 10.1136/gutjnl-2017-314759. Epub 2017 Aug 16. PMID: 28814485; PMCID: PMC5851874.	Included in systematic review and meta-analysis (Data requested and kindly provided by the authors)
64. Maes M, Kubera M, Leunis JC, Berk M. Increased IgA and IgM responses against gut commensals in chronic depression: further evidence for increased bacterial translocation or leaky gut. J Affect Disord. 2012 Dec 1;141(1):55-62. doi: 10.1016/j.jad.2012.02.023. Epub 2012 Mar 11. PMID: 22410503.	Excluded- Lack of data<br>(Possible duplicate)
65. Gomes C, Martinho FC, Barbosa DS, Antunes LS, Póvoa HCC, Baltus THL, Morelli NR, Vargas HO, Nunes SOV, Anderson G, Maes M. Increased Root Canal Endotoxin Levels are Associated with Chronic Apical Periodontitis, Increased Oxidative and Nitrosative Stress, Major Depression, Severity of Depression, and a Lowered Quality of Life. Mol Neurobiol. 2018 Apr;55(4):2814-2827. doi: 10.1007/s12035-017-0545-z. Epub 2017 Apr 28. PMID: 28455694.	Excluded- Lack of data
66. Simeonova D, Stoyanov D, Leunis JC, Carvalho AF, Kubera M, Murdjeva M, Maes M. Increased Serum Immunoglobulin Responses to Gut Commensal Gram-Negative Bacteria in Unipolar Major Depression and Bipolar Disorder Type 1, Especially When Melancholia Is Present. Neurotox Res. 2020 Feb;37(2):338-348. doi: 10.1007/s12640-019-00126-7. Epub 2019 Dec 4. PMID: 31802379.	Excluded- Lack of data (Possible duplicate)
67. Zhou Z, Guille C, Ogunrinde E, Liu R, Luo Z, Powell A, Jiang W. Increased systemic microbial translocation is associated with depression during early pregnancy. J Psychiatr Res. 2018 Feb;97:54-57. doi:	Included in systematic review<br>(Additional data requested but not received)

10.1016/j.jpsychires.2017.11.009. Epub 2017 Nov 22. PMID: 29179013; PMCID: PMC5742552.	
68. Lu H, Surkan PJ, Irwin MR, Treisman GJ, Breen EC, Sacktor N, Stall R, Wolinsky SM, Jacobson LP, Abraham AG. Inflammation and Risk of Depression in HIV: Prospective Findings From the Multicenter AIDS Cohort Study. Am J Epidemiol. 2019 Nov 1;188(11):1994-2003. doi: 10.1093/aje/kwz190. PMID: 31642472; PMCID: PMC6825834.	Excluded- Lack of data
69. Ballesio A, Micheli F, Baccini F, Zagaria A, Del Forno A, Fiori V, Palombelli G, Scalamonti S, Ruffa A, Magiotta A, Di Nardo G, Lombardo C. Inflammation as an aetiological trigger for depressive symptoms in a prospective cohort of patients with inflammatory bowel disease. J Psychosom Res. 2024 Feb;177:111592. doi: 10.1016/j.jpsychores.2024.111592. Epub 2024 Jan 10. PMID: 38217896.	Excluded- Lack of data
70. Rozing MP, Veerhuis R, Westendorp RGJ, Eikelenboom P, Stek M, Marijnissen RM, Oude Voshaar RC, Comijs HC, van Exel E. Inflammation in older subjects with early- and late-onset depression in the NESDO study: a cross-sectional and longitudinal case-only design. Psychoneuroendocrinology. 2019 Jan;99:20-27. doi: 10.1016/j.psyneuen.2018.08.029. Epub 2018 Aug 24. PMID: 30172071.	Excluded – Wrong focus (Focus different from the one of interest)
71. González-Moret R, Cebolla-Martí A, Almodóvar-Fernández I, Navarrete J, García-Esparza Á, Soria JM, Lisón JF. Inflammatory biomarkers and psychological variables to assess quality of life in patients with inflammatory bowel disease: a cross-sectional study. Ann Med. 2024 Dec;56(1):2357738. doi: 10.1080/07853890.2024.2357738. Epub 2024 May 31. PMID: 38819080; PMCID: PMC11146243.	Excluded- Lack of data
72. Osuna E, Baumgartner J, Wunderlin O, Emery S, Albermann M, Baumgartner N, Schmeck K, Walitza S, Strumberger M, Hersberger M, Zimmermann MB, Häberling I, Berger G, Herter-Aeberli I;	Included in systematic review and meta-analysis

Omega-3 Study Team. Iron status in Swiss adolescents with paediatric major depressive disorder and healthy controls: a matched case-control study. Eur J Nutr. 2024 Apr;63(3):951-963. doi: 10.1007/s00394-023-03313-7. Epub 2024 Jan 24. PMID: 38265750; PMCID: PMC10948461.	
73. Keohane J, O'Mahony C, O'Mahony L, O'Mahony S, Quigley EM, Shanahan F. Irritable bowel syndrome-type symptoms in patients with inflammatory bowel disease: a real association or reflection of occult inflammation? Am J Gastroenterol. 2010 Aug;105(8):1788, 1789-94; quiz 1795. doi: 10.1038/ajg.2010.156. Epub 2010 Apr 13. PMID: 20389294.	Excluded- Lack of data<br>(Correlation between HAD score and Fecal calprotectin)
74. Karaoulanis SE, Rizouli KA, Rizoulis AA, Angelopoulos NV. Lack of association of acute phase response proteins with hormone levels and antidepressant medication in perimenopausal depression. BMC Psychiatry. 2014 Jun 4;14:164. doi: 10.1186/1471-244X-14-164. PMID: 24894416; PMCID: PMC4084572.	Included in systematic review and meta-analysis
75. Ohlsson L, Gustafsson A, Lavant E, Suneson K, Brundin L, Westrin Å, Ljunggren L, Lindqvist D. Leaky gut biomarkers in depression and suicidal behavior. Acta Psychiatr Scand. 2019 Feb;139(2):185-193. doi: 10.1111/acps.12978. Epub 2018 Nov 1. Erratum in: Acta Psychiatr Scand. 2020 Nov;142(5):423. doi: 10.1111/acps.13223. PMID: 30347427; PMCID: PMC6587489.	Included in systematic review and meta-analysis
76. Bai S, Fang L, Xie J, Bai H, Wang W, Chen JJ. Potential Biomarkers for Diagnosing Major Depressive Disorder Patients with Suicidal Ideation. J Inflamm Res. 2021 Feb 22;14:495-503. doi: 10.2147/JIR.S297930. PMID: 33654420; PMCID: PMC7910095.	Included in systematic review and meta-analysis<br>(Additional data requested: after two attempts, the data were manually extracted using web-based tools)
77. Hirten RP, Danieletto M, Scheel R, Shervey M, Ji J, Hu L, Sauk J, Chang L, Arnrich B, Böttinger E, Dudley J, Keefer L, Sands BE. Longitudinal Autonomic Nervous System Measures Correlate With Stress and Ulcerative Colitis	Included in systematic review

Disease Activity and Predict Flare. Inflamm Bowel Dis. 2021 Oct 18;27(10):1576-1584. doi: 10.1093/ibd/izaa323. PMID: 33382065.	
78. Fairbrass KM, Gracie DJ, Ford AC. Longitudinal follow-up study: effect of psychological co-morbidity on the prognosis of inflammatory bowel disease. Aliment Pharmacol Ther. 2021 Aug;54(4):441-450. doi: 10.1111/apt.16454. Epub 2021 Jun 11. PMID: 34114664.	Excluded- Lack of data (Correlation between HADS-D score and Fecal calprotectin)
79. Kiecolt-Glaser JK, Wilson SJ, Bailey ML, Andridge R, Peng J, Jaremka LM, Fagundes CP, Malarkey WB, Laskowski B, Belury MA. Marital distress, depression, and a leaky gut: Translocation of bacterial endotoxin as a pathway to inflammation. Psychoneuroendocrinology. 2018 Dec;98:52-60. doi: 10.1016/j.psyneuen.2018.08.007. Epub 2018 Aug 4. PMID: 30098513; PMCID: PMC6260591.	Included in systematic review
80. Cai Y, Gong D, Xiang T, Zhang X, Pan J. Markers of intestinal barrier damage in patients with chronic insomnia disorder. Front Psychiatry. 2024 Mar 28;15:1373462. doi: 10.3389/fpsyt.2024.1373462. PMID: 38606411; PMCID: PMC11007705.	Included in systematic review
81. Bilello JA, Thurmond LM, Smith KM, Pi B, Rubin R, Wright SM, Taub F, Henry ME, Shelton RC, Papakostas GI. MDDScore: confirmation of a blood test to aid in the diagnosis of major depressive disorder. J Clin Psychiatry. 2015 Feb;76(2):e199-206. doi: 10.4088/JCP.14m09029. PMID: 25742207.	Excluded- Lack of data (Correlation between HDRS-17 score and α1 antitrypsin)
82. Goodhand JR, Wahed M, Mawdsley JE, Farmer AD, Aziz Q, Rampton DS. Mood disorders in inflammatory bowel disease: relation to diagnosis, disease activity, perceived stress, and other factors. Inflamm Bowel Dis. 2012 Dec;18(12):2301-9. doi: 10.1002/ibd.22916. Epub 2012 Feb 22. PMID: 22359369.	Excluded- Lack of data (Correlation between HADS-D score and Fecal calprotectin)
83. Cohen-Mekelburg S, Goldstein CA, Rizvydeen M, Fayyaz Z, Patel PJ,	Excluded- Lack of data

Berinstein JA, Bishu S, Cushing-Damm KC, Kim HM, Burgess HJ. Morning light treatment for inflammatory bowel disease: a clinical trial. BMC Gastroenterol. 2024 May 22;24(1):179. doi: 10.1186/s12876-024-03263-2. PMID: 38778264; PMCID: PMC11110384.	(Correlation between PHQ-9 score and Fecal calprotectin)
84. Riggott C, Fairbrass KM, Black CJ, Gracie DJ, Ford AC. Novel symptom clusters predict disease impact and healthcare utilisation in inflammatory bowel disease: Prospective longitudinal follow-up study. Aliment Pharmacol Ther. 2023 Dec;58(11-12):1163-1174. doi: 10.1111/apt.17735. Epub 2023 Oct 4. PMID: 37792347.	Excluded- Lack of data<br>(Correlation between HADS-D score and Fecal calprotectin)
85. Lundgren D, Rutegård J, Eklöf V, Palmqvist R, Karling P. Patients with longstanding ulcerative colitis in remission do not have more irritable bowel syndrome-like symptoms than controls. BMC Gastroenterol. 2016 Nov 24;16(1):139. doi: 10.1186/s12876-016-0553-x. PMID: 27881072; PMCID: PMC5121960.	Included in systematic review (Correlation between HADS-D score and Fecal calprotectin levels, as well as other variables, was performed by our team based on the dataset provided as an Additional file by the Authors)
86. Zhang L, Liu C, Li Y, Wu Y, Wei Y, Zeng D, He S, Huang J, Li H. Plasma biomarker panel for major depressive disorder by quantitative proteomics using ensemble learning algorithm: A preliminary study. Psychiatry Res. 2023 May;323:115185. doi: 10.1016/j.psychres.2023.115185. Epub 2023 Mar 28. PMID: 37003170.	Excluded – Wrong focus (Focus different from the one of interest)
87. Gracie DJ, Williams CJ, Sood R, Mumtaz S, Bholah MH, Hamlin PJ, Ford AC. Poor Correlation Between Clinical Disease Activity and Mucosal Inflammation, and the Role of Psychological Comorbidity, in Inflammatory Bowel Disease. Am J Gastroenterol. 2016 Apr;111(4):541-51. doi: 10.1038/ajg.2016.59. Epub 2016 Mar 22. PMID: 27002800.	Excluded- Lack of data<br>(Correlation between HADS-D score and Fecal calprotectin)
88. Ahmed M, Pu A, Jencks K, Bishu S, Higgins P, Chey WD, Rao K, Lee A. Predictors of irritable bowel syndrome-like symptoms in quiescent inflammatory bowel disease. Neurogastroenterol Motil. 2024	Excluded – Wrong focus (Focus different from the one of interest)

Jun;36(6):e14809. doi: 10.1111/nmo.14809. Epub 2024 Apr 23. PMID: 38651743.	
89. Cho HJ, Eisenberger NI, Olmstead R, Breen EC, Irwin MR. Preexisting mild sleep disturbance as a vulnerability factor for inflammation-induced depressed mood: a human experimental study. Transl Psychiatry. 2016 Mar 8;6(3):e750. doi: 10.1038/tp.2016.23. PMID: 26954978; PMCID: PMC4872448.	Excluded – Wrong focus (Focus different from the one of interest)
90. Goldsmith DR, Bekhbat M, Le NA, Chen X, Woolwine BJ, Li Z, Haroon E, Felger JC. Protein and gene markers of metabolic dysfunction and inflammation together associate with functional connectivity in reward and motor circuits in depression. Brain Behav Immun. 2020 Aug;88:193-202. doi: 10.1016/j.bbi.2020.05.013. Epub 2020 May 5. PMID: 32387344; PMCID: PMC7415617.	Excluded – Wrong focus (Focus different from the one of interest)
91. Chen C, Hu Y, Dong XZ, Zhou XJ, Mu LH, Liu P. Proteomic Analysis of the Antidepressant Effects of Shen-Zhi-Ling in Depressed Patients: Identification of Proteins Associated with Platelet Activation and Lipid Metabolism. Cell Mol Neurobiol. 2018 Jul;38(5):1123-1135. doi: 10.1007/s10571-018-0582-9. Epub 2018 Mar 21. PMID: 29564590.	Excluded- Lack of data (Correlation between HADS-D score and alpha-1-antitrypsin among patients at baseline)
92. Reininghaus EZ, Platzer M, Kohlhammer-Dohr A, Hamm C, Mörkl S, Bengesser SA, Fellendorf FT, Lahousen-Luxenberger T, Leitner-Afschar B, Schöggl H, Amberger-Otti D, Wurm W, Queissner R, Birner A, Falzberger VS, Painold A, Fitz W, Wagner-Skacel J, Brunnmayr M, Rieger A, Maget A, Unterweger R, Schwalsberger K, Reininghaus B, Lenger M, Bastiaanssen TFS, Dalkner N. PROVIT: Supplementary Probiotic Treatment and Vitamin B7 in Depression-A Randomized Controlled Trial. Nutrients. 2020 Nov 8;12(11):3422. doi: 10.3390/nu12113422. PMID: 33171595; PMCID: PMC7695208.	Excluded- Lack of data (Correlation between HAM-D/BDI-II scores and Zonuline levels among participants at baseline)

93. Chen HM, Kuo PH, Hsu CY, Chiu YH, Liu YW, Lu ML, Chen CH. Psychophysiological Effects of Lactobacillus plantarum PS128 in Patients with Major Depressive Disorder: A Preliminary 8-Week Open Trial. Nutrients. 2021 Oct 22;13(11):3731. doi: 10.3390/nu13113731. PMID: 34835987; PMCID: PMC8618644.	Excluded- Lack of data<br>(Correlation between HAMD-17 score and I-FABP and Zonuline levels among participants at baseline)
94. Tersigni C, D'Ippolito S, Di Nicuolo F, Marana R, Valenza V, Masciullo V, Scaldaferri F, Malatacca F, de Waure C, Gasbarrini A, Scambia G, Di Simone N. Recurrent pregnancy loss is associated to leaky gut: a novel pathogenic model of endometrium inflammation? J Transl Med. 2018 Apr 17;16(1):102. doi: 10.1186/s12967-018-1482-y. Erratum in: J Transl Med. 2019 Mar 15;17(1):83. doi: 10.1186/s12967-019-1823-5. PMID: 29665864; PMCID: PMC5905157.	Excluded- Lack of data<br>(Correlation between Zung Self-Rating Depression Scale (Z-SDS) score and I-lipopolysaccharide (LPS) levels among participants at baseline)
95. Mobeen F, Edgar RG, Pye A, Stockley RA, Turner AM. Relationship between Depression and Anxiety, Health Status and Lung Function in Patients with Alpha-1 Antitrypsin Deficiency. COPD. 2021 Dec;18(6):621-629. doi: 10.1080/15412555.2021.1991904. Epub 2021 Oct 22. PMID: 34676796.	Excluded – Wrong focus (Focus different from the one of interest)
96. Fairbrass KM, Gracie DJ, Ford AC. Relative Contribution of Disease Activity and Psychological Health to Prognosis of Inflammatory Bowel Disease During 6.5 Years of Longitudinal Follow-Up. Gastroenterology. 2022 Jul;163(1):190-203.e5. doi: 10.1053/j.gastro.2022.03.014. Epub 2022 Mar 23. PMID: 35339461.	Excluded – Wrong focus (Focus different from the one of interest)
97. Tasson L, Zingone F, Barberio B, Valentini R, Ballotta P, Ford AC, Scarpa M, Angriman I, Fassan M, Savarino E. Sarcopenia, severe anxiety and increased C-reactive protein are associated with severe fatigue in patients with inflammatory bowel diseases. Sci Rep. 2021 Jul 27;11(1):15251. doi: 10.1038/s41598-021-94685-5. PMID: 34315951; PMCID: PMC8316564.	Excluded- Lack of data<br>(Correlation between HADS-D score and Fecal Calprotectin among patients)

98. Hochman E, Taler M, Flug R, Gur S, Dar S, Bormant G, Blattberg D, Nitzan U, Krivoy A, Weizman A. Serum claudin-5 levels among patients with unipolar and bipolar depression in relation to the pro-inflammatory cytokine tumor necrosis factor-alpha levels. Brain Behav Immun. 2023 Mar;109:162-167. doi: 10.1016/j.bbi.2023.01.015. Epub 2023 Jan 24. PMID: 36706845.	Excluded – Wrong focus (Focus different from the one of interest)
99. Kılıç F, Işık Ü, Kumbul Doğuç D. Serum Claudin-5, but not Zonulin, May Be Associated with Obsessive-Compulsive Disorder. Psychiatr Danub. 2022 Summer;34(2):273-278. doi: 10.24869/psyd.2022.273. PMID: 35772137.	Excluded- Lack of data<br>(Correlation between HDRS score and Zonulin levels)
100. Brys ADH, Di Stasio E, Lenaert B, Sanguinetti M, Picca A, Calvani R, Marzetti E, Gambaro G, Bossola M. Serum interleukin-6 and endotoxin levels and their relationship with fatigue and depressive symptoms in patients on chronic haemodialysis. Cytokine. 2020 Jan;125:154823. doi: 10.1016/j.cyto.2019.154823. Epub 2019 Sep 18. PMID: 31541903.	Included in systematic review
101. Kozłowska E, Wysokiński A, Brzezińska-Błaszczyk E. Serum levels of peptide cathelicidin LL-37 in elderly patients with depression. Psychiatry Res. 2017 Sep;255:156-160. doi: 10.1016/j.psychres.2017.05.036. Epub 2017 May 22. PMID: 28550757.	Excluded – Wrong focus (Focus different from the one of interest)
102. Aydoğan Avşar P, Işık Ü, Aktepe E, Kılıç F, Doğuç DK, Büyükbayram Hİ. Serum zonulin and claudin-5 levels in children with attention-deficit/hyperactivity disorder. Int J Psychiatry Clin Pract. 2021 Mar;25(1):49-55. doi: 10.1080/13651501.2020.1801754. Epub 2020 Aug 6. PMID: 32757874.	Excluded- Lack of data<br>(Correlation between RCADS-CV depression Subscale score and Zonulin levels)
103. Işık Ü, Aydoğan Avşar P, Aktepe E, Doğuç DK, Kılıç F, Büyükbayram Hİ. Serum zonulin and claudin-5 levels in children with obsessive-compulsive disorder. Nord J Psychiatry. 2020 May-Jul;74(5):346-351. doi: 10.1080/08039488.2020.1715474. Epub 2020 Jan 21. PMID: 31961248.	Included in systematic review

104. Kılıç F, Işık Ü, Demirdaş A, Doğuç DK, Bozkurt M. Serum zonulin and claudin-5 levels in patients with bipolar disorder. J Affect Disord. 2020 Apr 1;266:37-42. doi: 10.1016/j.jad.2020.01.117. Epub 2020 Jan 23. PMID: 32056901.	Included in systematic review
105. Maget A, Dalkner N, Hamm C, Bengesser SA, Fellendorf FT, Platzer M, Queissner R, Birner A, Lenger M, Mörkl S, Kohlhammer-Dohr A, Rieger A, Seidl M, Mendel L, Färber T, Wetzlmair L, Schwalsberger K, Amberger-Otti DV, Schöggl H, Lahousen T, Leitner-Afschar B, Unterweger R, Zelzer S, Mangge H, Reininghaus EZ. Sex differences in zonulin in affective disorders and associations with current mood symptoms. J Affect Disord. 2021 Nov 1;294:441-446. doi: 10.1016/j.jad.2021.07.021. Epub 2021 Jul 21. PMID: 34320451.	Included in systematic review and meta-analysis
106. Meinitzer S, Baranyi A, Holasek S, Schnedl WJ, Zelzer S, Mangge H, Herrmann M, Meinitzer A, Enko D. Sex-Specific Associations of Trimethylamine-N-Oxide and Zonulin with Signs of Depression in Carbohydrate Malabsorbers and Nonmalabsorbers. Dis Markers. 2020 Jan 6;2020:7897240. doi: 10.1155/2020/7897240. PMID: 31998418; PMCID: PMC6969987.	Included in systematic review
107. Prospero L, Riezzo G, Linsalata M, Orlando A, D'Attoma B, Di Masi M, Martulli M, Russo F. Somatization in patients with predominant diarrhoea irritable bowel syndrome: the role of the intestinal barrier function and integrity. BMC Gastroenterol. 2021 May 22;21(1):235. doi: 10.1186/s12876-021-01820-7. PMID: 34022802; PMCID: PMC8141183.	Excluded- Lack of data<br>(Correlation between depression levels and IFABP-2, Faecal Zonulin, Serum Zonulin, LPS levels)
108. Varanoske AN, McClung HL, Sepowitz JJ, Halagarda CJ, Farina EK, Berryman CE, Lieberman HR, McClung JP, Pasiakos SM, Philip Karl J. Stress and the gut-brain axis: Cognitive performance, mood state, and biomarkers of blood-brain barrier and intestinal permeability following severe	Included in systematic review

physical and psychological stress. Brain Behav Immun. 2022 Mar;101:383-393. doi: 10.1016/j.bbi.2022.02.002. Epub 2022 Feb 5. PMID: 35131441.	
109. Berrill JW, Green JT, Hood K, Campbell AK. Symptoms of irritable bowel syndrome in patients with inflammatory bowel disease: examining the role of sub-clinical inflammation and the impact on clinical assessment of disease activity. Aliment Pharmacol Ther. 2013 Jul;38(1):44-51. doi: 10.1111/apt.12335. Epub 2013 May 13. PMID: 23668698.	Excluded – Wrong focus (Focus different from the one of interest)
110. Louzada ER, Ribeiro SML. Synbiotic supplementation, systemic inflammation, and symptoms of brain disorders in elders: A secondary study from a randomized clinical trial. Nutr Neurosci. 2020 Feb;23(2):93-100. doi: 10.1080/1028415X.2018.1477349. Epub 2018 May 23. PMID: 29788823.	Included in systematic review
111. Dickerson F, Adamos M, Katsafanas E, Khushalani S, Origoni A, Savage C, Schweinfurth L, Stallings C, Sweeney K, Alaedini A, Uhde M, Severance E, Wilcox HC, Yolken R. The association between immune markers and recent suicide attempts in patients with serious mental illness: A pilot study. Psychiatry Res. 2017 Sep;255:8-12. doi: 10.1016/j.psychres.2017.05.005. Epub 2017 May 9. PMID: 28505469.	Included in systematic review<br>(Additional data requested but not received: LPS_IgA levels and C-SSRS scores among n.20 patients with recent attempt group and n.95 patients with no attempt group; LPS_IgA levels and BPRS scores among 48 patients with MDD and 72 non-psychiatric controls).
112. Deleemans JM, Chleilat F, Reimer RA, Henning JW, Baydoun M, Piedalue KA, McLennan A, Carlson LE. The chemo-gut study: investigating the long-term effects of chemotherapy on gut microbiota, metabolic, immune, psychological and cognitive parameters in young adult Cancer survivors; study protocol. BMC Cancer. 2019 Dec 23;19(1):1243. doi: 10.1186/s12885-019-6473-8. PMID: 31870331; PMCID: PMC6927187.	Excluded- Lack of data<br>(Correlation between PROMIS - Ca Item Bank v1.0 - Emotional Distress - Depression questionnaire scores and LPS levels)
113. Gerbarg PL, Jacob VE, Stevens L, Bosworth BP, Chabouni F, DeFilippis EM, Warren R, Trivellas M, Patel PV, Webb CD, Harbus MD, Christos PJ, Brown RP, Scherl EJ. The Effect of Breathing, Movement, and Meditation	Excluded- Lack of data<br>(Correlation between BDI scores and fecal calprotectin levels at baseline)

on Psychological and Physical Symptoms and Inflammatory Biomarkers in Inflammatory Bowel Disease: A Randomized Controlled Trial. Inflamm Bowel Dis. 2015 Dec;21(12):2886-96. doi: 10.1097/MIB.0000000000000568. PMID: 26426148.	
114. Jaehne EJ, Corrigan F, Toben C, Jawahar MC, Baune BT. The effect of the antipsychotic drug quetiapine and its metabolite norquetiapine on acute inflammation, memory and anhedonia. Pharmacol Biochem Behav. 2015 Aug;135:136-44. doi: 10.1016/j.pbb.2015.05.021. Epub 2015 Jun 3. PMID: 26047769.	Excluded- Lack of data<br>(Correlation between BDI-II scores and LPS levels at baseline)
115. Kiecolt-Glaser JK, Wilson SJ, Shrout MR, Madison AA, Andridge R, Peng J, Malarkey WB, Bailey MT. The gut reaction to couples' relationship troubles: A route to gut dysbiosis through changes in depressive symptoms. Psychoneuroendocrinology. 2021 Mar;125:105132. doi: 10.1016/j.psyneuen.2021.105132. Epub 2021 Jan 14. PMID: 33486306; PMCID: PMC7904664.	Included in narrative review
116. Maes M, Kubera M, Leunis JC. The gut-brain barrier in major depression: intestinal mucosal dysfunction with an increased translocation of LPS from gram negative enterobacteria (leaky gut) plays a role in the inflammatory pathophysiology of depression. Neuro Endocrinol Lett. 2008 Feb;29(1):117-24. PMID: 18283240.	Included in systematic review and meta-analysis
117. Mules TC, Swaminathan A, Hirschfeld E, Borichevsky G, Frampton C, Day AS, Gearry RB. The impact of disease activity on psychological symptoms and quality of life in patients with inflammatory bowel disease-results from the Stress, Anxiety and Depression with Disease Activity (SADD) Study. Aliment Pharmacol Ther. 2022 Jan;55(2):201-211. doi: 10.1111/apt.16616. Epub 2021 Sep 29. PMID: 34587655.	Excluded- Lack of data<br>(Correlation between PHQ-9 scores and fecal calprotectin levels at baseline)
118. Rog J, Karakuła M, Rząd Z, Fitowska A, Brzezińska A, Karakula-	Included in narrative review

Juchnowicz H. The Importance of Adverse Childhood Experiences in Depressive Symptoms and Their Biological Consequences in Healthy Adults: Results of a Polish University Student Study. J Clin Med. 2023 Nov 14;12(22):7093. doi: 10.3390/jcm12227093. PMID: 38002705; PMCID: PMC10671862.	(Lack of data on levels of Lipopolysaccharide Binding Protein, Occludin, Tight Junction Protein 1, and Anti-zonulin antibody in cases and controls).
119. Roomruangwong C, Carvalho AF, Geffard M, Maes M. The menstrual cycle may not be limited to the endometrium but also may impact gut permeability. Acta Neuropsychiatr. 2019 Dec;31(6):294-304. doi: 10.1017/neu.2019.30. Epub 2019 Oct 14. PMID: 31364524.	Excluded- Lack of data<br>(Correlation between Daily Record of Severity of Problems – Depression dimension scores and peak IgA levels at baseline)
120. Vasant DH, Nigam GB, Bate S, Hamdy S, Limdi JK. The prevalence and burden of Rome IV faecal incontinence in ulcerative colitis: A cross-sectional study. Aliment Pharmacol Ther. 2023 Jul;58(1):26-34. doi: 10.1111/apt.17502. Epub 2023 Apr 3. PMID: 37012703.	Excluded- Lack of data<br>(Correlation between HADS-D scores and fecal calprotectin levels at baseline)
121. Drinčić T, van Dalfsen JH, Kamphuis J, Jentsch MC, van Belkum SM, Meddens MJM, Penninx BWJH, Schoevers RA. The Relationship between Insomnia and the Pathophysiology of Major Depressive Disorder: An Evaluation of a Broad Selection of Serum and Urine Biomarkers. Int J Mol Sci. 2023 May 8;24(9):8437. doi: 10.3390/ijms24098437. PMID: 37176140; PMCID: PMC10179282.	Excluded- Lack of data
122. Maes M, Simeonova D, Stoyanov D, Leunis JC. Upregulation of the nitrosylome in bipolar disorder type 1 (BP1) and major depression, but not BP2: Increased IgM antibodies to nitrosylated conjugates are associated with indicants of leaky gut. Nitric Oxide. 2019 Oct 1;91:67-76. doi: 10.1016/j.niox.2019.07.003. Epub 2019 Jul 16. PMID: 31323278.	Included in systematic review and meta-analysis
123. Olmedo Martín RV, González Molero I, Olveira Fuster G, Amo Trillo V, Jiménez Pérez M. Vitamin D deficiency in outpatients with inflammatory bowel disease: prevalence	Excluded- Lack of data<br>(Correlation between HAM-D scores and Fecal Calprotectin levels at baseline)

and association with clinical-biological activity. Rev Esp Enferm Dig. 2019 Jan;111(1):46-54. doi: 10.17235/reed.2018.5714/2018. PMID: 30284908.	

Additional from references

124. Jakobsson J, Bjerke M, Sahebi S, Isgren A, Ekman CJ, Sellgren C, Olsson B, Zetterberg H, Blennow K, Pålsson E, Landén M. Monocyte and microglial activation in patients with mood-stabilized bipolar disorder. J Psychiatry Neurosci. 2015 Jul;40(4):250-8. doi: 10.1503/jpn.140183. PMID: 25768030; PMCID: PMC4478058.	Excluded - Wrong focus (Focus different from the one of interest)
125. Tanaka T, Matsuda T, Hayes LN, Yang S, Rodriguez K, Severance EG, Yolken RH, Sawa A, Eaton WW. Infection and inflammation in schizophrenia and bipolar disorder. Neurosci Res. 2017 Feb;115:59-63. doi: 10.1016/j.neures.2016.11.002. Epub 2016 Nov 14. PMID: 27856235.	Excluded - Wrong focus (Focus different from the one of interest)
126. Severance EG, Gressitt KL, Stallings CR, Origoni AE, Khushalani S, Leweke FM, Dickerson FB, Yolken RH. Discordant patterns of bacterial translocation markers and implications for innate immune imbalances in schizophrenia. Schizophr Res. 2013 Aug;148(1-3):130-7. doi: 10.1016/j.schres.2013.05.018. Epub 2013 Jun 6. PMID: 23746484; PMCID: PMC3732507.	Excluded - Wrong focus (Focus different from the one of interest)
127. Čiháková D, Eaton WW, Talor MV, Harkus UH, Demyanovich H, Rodriguez K, Feldman S, Kelly DL. Gut permeability and mimicry of the Glutamate Ionotropic Receptor NMDA type Subunit Associated with protein 1 (GRINA) as potential mechanisms related to a subgroup of people with schizophrenia with elevated antigliadin antibodies (AGA IgG). Schizophr Res. 2019 Jun;208:414-419. doi: 10.1016/j.schres.2019.01.007. Epub 2019 Jan 24. PMID: 30685393.	Excluded - Wrong focus (Focus different from the one of interest)from the one of interest

128. Mørch RH, Dieset I, Færden A, Reponen EJ, Hope S, Hoseth EZ, Gardsjord ES, Aas M, Iversen T, Joa I, Morken G, Agartz I, Melle I, Aukrust P, Djurovic S, Ueland T, Andreassen OA. Inflammatory markers are altered in severe mental disorders independent of comorbid cardiometabolic disease risk factors. Psychol Med. 2019 Jul;49(10):1749-1757. doi: 10.1017/S0033291718004142. Epub 2019 Jan 28. Erratum in: Psychol Med. 2019 Jul;49(10):1758. doi: 10.1017/S0033291719000291. PMID: 30688187.	Excluded - Wrong focus (Focus different from the one of interes; no specific data for only depressed/suicidal patients)
129. Severance EG, Gressitt KL, Yang S, Stallings CR, Origoni AE, Vaughan C, Khushalani S, Alaedini A, Dickerson FB, Yolken RH. Seroreactive marker for inflammatory bowel disease and associations with antibodies to dietary proteins in bipolar disorder. Bipolar Disord. 2014 May;16(3):230-40. doi: 10.1111/bdi.12159. Epub 2013 Dec 6. PMID: 24313887; PMCID: PMC4075657.	Excluded - Wrong focus (Focus different from the one of interest)from the one of interest

Additional from free search

130. Yee, J.Y., Nurjono, M., Ng, W.Y., Teo, S.R., Lee, T.-S. & Lee, J. (2017) Peripheral blood gene expression of acute phase proteins in people with first episode psychosis. *Brain, behavior, and immunity*. 65, 337–341. doi:10.1016/j.bbi.2017.06.006.	Included in systematic review
131. Vincent Chin-Hung Chen, Shu-I Wu. An exploratory analysis on the association between suicidal ideation and the microbiome in patients with or without major depressive disorder, 21 March 2024, PREPRINT (Version 1) available at Research Square [https://doi.org/10.21203/rs.3.rs-4119552/v1]	Included in systematic review and meta-analysis
132. Zhong J, Chen J, Cao M, Fang L, Wang Z, Liao J, Chen D, Zhang X, Guo J, Zhao L, Zhou C. Elevated plasma intestinal fatty acid binding protein and aberrant lipid metabolism	Included in systematic review and meta-analysis

predict post-stroke depression. Heliyon. 2022 Nov 23;8(11):e11848. doi: 10.1016/j.heliyon.2022.e11848. PMID: 36468110; PMCID: PMC9713332.	
133. Wang L, Chunyou C, Zhu J, Bao X, Tao X. Prediction of post-stroke depression with combined blood biomarkers IL-6, TNF-a, and fatty acid binding protein: A prospective study. J Med Biochem. 2023 Oct 27;42(4):638-644. doi: 10.5937/jomb0-43904. PMID: 38084247; PMCID: PMC10710798.	Included in systematic review and meta-analysis
134. Iordache MM, Belu AM, Vlad SE, Aivaz KA, Dumitru A, Tocia C, Dumitru E. Calprotectin, Biomarker of Depression in Patients with Inflammatory Bowel Disease? Medicina (Kaunas). 2023 Jul 3;59(7):1240. doi: 10.3390/medicina59071240. PMID: 37512053; PMCID: PMC10383955	Excluded (Duplicate, same population that: 30. Iordache MM, Tocia C, Aschie M, Dumitru A, Manea M, Cozaru GC, Petcu L, Vlad SE, Dumitru E, Chisoi A. Intestinal Permeability and Depression in Patients with Inflammatory Bowel Disease. J Clin Med. 2022 Aug 30;11(17):5121. doi: 10.3390/jcm11175121. PMID: 36079050; PMCID: PMC9457405..
